# Gold-catalyzed regioselective oxidation of terminal allenes: formation of α-methanesulfonyloxy methyl ketones

**DOI:** 10.3762/bjoc.7.69

**Published:** 2011-05-11

**Authors:** Yingdong Luo, Guozhu Zhang, Erik S Hwang, Thomas A Wilcoxon, Liming Zhang

**Affiliations:** 1 Department of Chemistry and Biochemistry, University of California, Santa Barbara, California, 93106, USA

**Keywords:** allene, catalysis, gold, oxidation, regioselectivity

## Abstract

Synthetically useful α-methanesulfonyloxy methyl ketones are readily prepared in one-step from terminal allenes in fair to good yields. The chemistry relies on a gold-catalyzed intermolecular oxidation of the 1,2-diene unit using 3,5-dichloropyridine *N*-oxide as the oxidant. The reaction tolerates a range of functional groups and shows excellent regioselectivity.

## Introduction

**Scheme 1:**
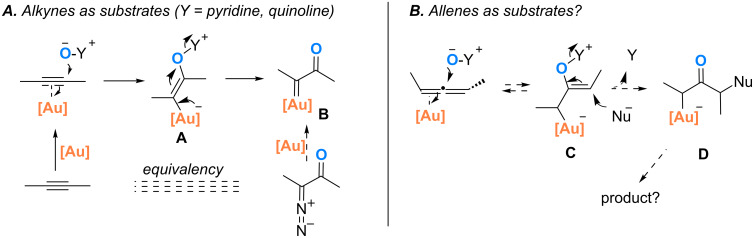
Gold-catalyzed intermolecular oxidation of alkynes and allenes.

While alkynes are the most studied substrates in homogeneous gold catalysis [1–9], allenes [10] occupy a not-so-distant second place, and many versatile transformations have been developed either using allenes as substrates [11–17] or via allenes generated in situ [18–21]. We have recently shown that highly reactive gold carbenes can be generated from alkynes via gold-promoted intermolecular oxidation by pyridine/quinoline *N*-oxides [22–25], making benign alkynes effective surrogates of toxic and potentially explosive α-diazo ketones (Scheme 1A). Synthetically useful structures such as oxetan-3-ones [22], dihydrofuran-3-ones [23], azetidin-3-ones [24] and α,β-unsaturated ketones [25] are readily accessed via these gold carbene intermediates. This led us to consider whether or not allenes could also be oxidized by these *N*-oxides in the presence of gold catalysts. As shown in Scheme 1B, intermediate **C**, likely formed via an initial nucleophilic attack of a gold-activated allene, cannot undergo elimination in the same way as intermediate **A**, hence gold carbene intermediate **B** would not be formed. While **C** may revert back to the allene substrate, we suspect that a S_N_2'-type reaction by an external nucleophile could facilitate the fragmentation of the O–Y bond, ultimately leading to useful products via intermediate **D**. Herein we report our preliminary studies, which led to a facile synthesis of α-methanesulfonyloxy methyl ketones.

## Results and Discussion

Initially, trideca-1,2-diene (**1a**) was treated with commercially available pyridine *N*-oxide in the presence of MsOH (5 equiv) and Ph_3_PAuNTf_2_ (5 mol %) in DCE at room temperature. Consumption of **1a** was initially observed and was complete in two days. A relatively polar compound was detected and subsequently isolated (Table 1, entry 1). NMR and MS analysis showed it to be the α-methanesulfonyloxy ketone **2a**. Interestingly, its regioisomer (i.e., **3**) was not observed, suggesting excellent regioselectivity in terms of the MsO delivery. The reaction time was shortened to 8 h by increasing the reaction temperature (entry 2). Attempts to increase the reaction efficiency by varying the *N*-oxide (entries 3–5) revealed that 3,5-dichloropyridine *N*-oxide was a superior oxidant, and **2a** was formed in 75% NMR yield. While less reactive but bulky gold catalysts, such as IPrAuNTf_2_ (entry 6) and Cy-JohnPhosAuNTf_2_ (entry 7), did not fare as well as Ph_3_PAuNTf_2_, the more Lewis acidic (4-CF_3_Ph)_3_PAuNTf_2_ was better, and **2a** was formed in 77% isolated yield (entry 8). A decrease in the amount of MsOH was counterproductive (entry 9), whilst no desired product was observed in the absence of a gold catalyst (entry 10).

**Table 1:** Initial studies and condition optimization^a^*.*

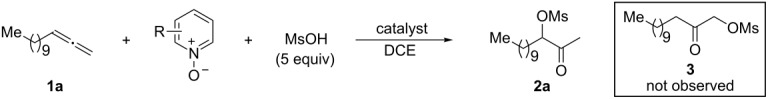				

Entry	Catalyst (5 mol %)	*N*-Oxide (2 equiv)	Reaction conditions	Yield^b^ (%)

1	Ph_3_PAuNTf_2_	pyridine *N*-oxide	rt, 2 d	46
2	Ph_3_PAuNTf_2_	pyridine *N*-oxide	40 °C, 8 h	52
3	Ph_3_PAuNTf_2_	quinoline *N*-oxide	40 °C, 8 h	51/6^c^
4	Ph_3_PAuNTf_2_	2-bromopyridine *N*-oxide	40 °C, 8 h	44/10^c^
5	Ph_3_PAuNTf_2_	3,5-dichloropyridine *N*-oxide	40 °C, 8 h	75
6	IPrAuNTf_2_	3,5-dichloropyridine *N*-oxide	40 °C, 8 h	10/53^c^
7	Cy-JohnPhosAuNTf_2_	3,5-dichloropyridine *N*-oxide	40 °C, 8 h	47/7^c^
8	(4-CF_3_Ph)_3_PAuNTf_2_	3,5-dichloropyridine *N*-oxide	40 °C, 8 h	80(77^d^)
9^e^	(4-CF_3_Ph)_3_PAuNTf_2_	3,5-dichloropyridine *N*-oxide	40 °C, 8 h	55/13^c^
10	—	3,5-dichloropyridine *N*-oxide	40 °C, 8 h	—

^a^[**1a**] = 0.05 M; ^b^determined by ^1^H NMR using diethyl phthalate as the external standard; ^c^unreacted starting material; ^d^isolated yield; ^e^2.5 equiv of MsOH.

With the optimized reaction conditions established (Table 1, entry 8), the scope of this chemistry was studied. As shown in Table 2, remote functional groups were readily tolerated. For example, good yields were obtained in the presence of a distal acetoxy (entry 1) or benzoyloxy (entry 2) group; moreover, reactive tosyloxy and mesyloxy groups were also tolerated (entries 3 and 4). A chloro (entry 5), a benzyloxy (entry 6), a protected amino (entry 7) and a phenyl group (entry 8) were also allowed, and the corresponding α-functionalized ketones were isolated in useful yields. Besides linear allenes, exocyclic allenes such as **1j** and **1k** were also suitable substrates and gave mesylates **2j** and **2k** in 72% and 59% yield, respectively (entries 9 and 10).

**Table 2:** Reaction scope^a^*.*

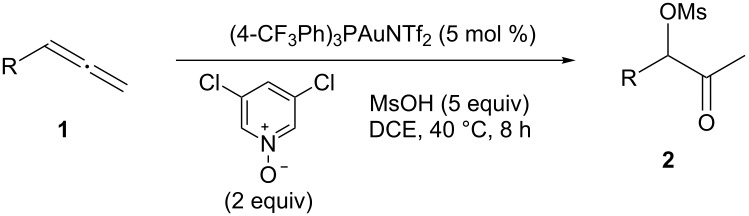					

Entry	Allene		Products		Yield^b^

1	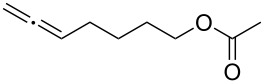	**1b**	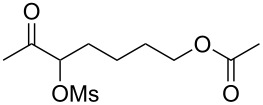	**2b**	79%
2	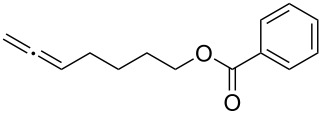	**1c**	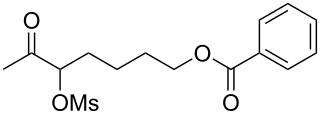	**2c**	75%
3	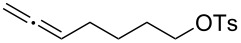	**1d**	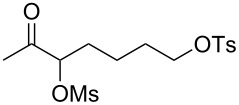	**2d**	80%
4	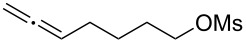	**1e**	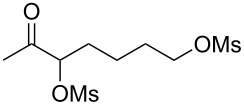	**2e**	73%
5	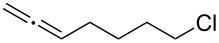	**1f**	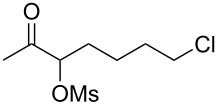	**2f**	63%
6	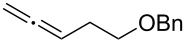	**1g**	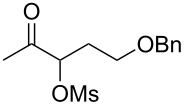	**2g**	61%
7	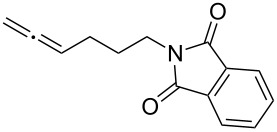	**1h**	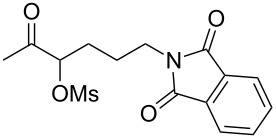	**2h**	76%
8	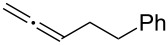	**1i**	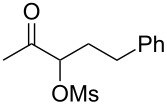	**2i**	60%
9	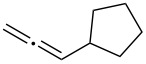	**1j**	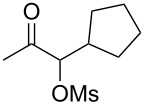	**2j**	72%
10	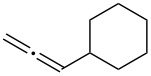	**1k**	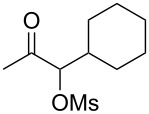	**2k**	59%

^a^[**1**] = 0.05 M; ^b^isolated yield.

**Scheme 2:**
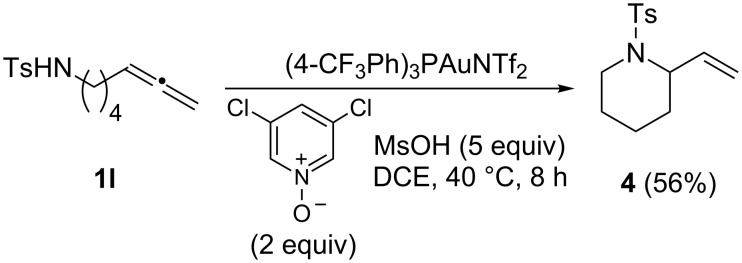
A side reaction from **1l**.

Some substrates, however, did not participate in this reaction effectively. For example, allenes derived by replacing the acetoxy group of **1b** with a free OH or an OTBS group did not lead to the desired products. Presumably, the nucleophilic OH group in the substrate, or one generated via acidic desilylation, interfered with the reaction. This reasoning was supported by the isolation of piperidine **4** upon subjecting **1l** to the optimized reaction conditions (Scheme 2). In addition, allenylbenzene was not a good substrate, and <10% of the desired ketone was detected by NMR. Somewhat surprisingly, pentadeca-7,8-diene [26], an internal allene, did not participate in the reaction.

It is of note that α-methanesulfonyloxy ketones are versatile synthetic intermediates that can undergo various reactions [27], including substitution [28], elimination [29], the formation of zinc homoenolates [30], the formation of cyclopropane rings under photo-irradiation [31–33], the formation of aminoimidazoles [34], the generation of cyclopropanone–oxyallyl intermediates [35], and ring contraction [36]. Their direct synthesis from corresponding ketones can be realized via oxidation by using either CuO/MsOH [37–38] or PhI(OH)OMs [39]. However, the former method uses stoichiometric amounts of copper, whilst the latter suffers from low regioselectivities. This gold-catalyzed approach offers an attractive alternative that is highly regioselective, catalytic on gold and takes place under relatively mild reaction conditions.

**Scheme 3:**
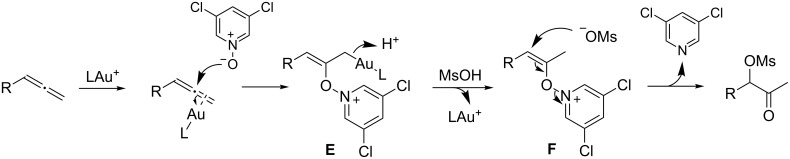
A proposed reaction mechanism.

The mechanism of this highly regioselective gold-catalyzed oxidation of allenes is proposed in Scheme 3. The first step, as in the case of alkyne oxidation [22–23,25], is probably an attack by the pyridine *N*-oxide on the gold-activated allene. Selective reaction at the terminal C–C double bond should occur due to steric preference. The allyl gold intermediate **E** can then undergo protonation to form intermediate **F** with MsO^−^ as the counter anion. An S_N_2'-type substitution by the anion would afford the observed product. This substitution is facilitated by the fragmentation of the weak N–O bond and the annihilation of the charges.

## Conclusion

We have successfully realized the first gold-catalyzed intermolecular oxidation of allenes. With 3,5-dichloropyridine *N*-oxide as the oxidant and in the presence of MsOH, α-methanesulfonyloxy methyl ketones are formed in one step in fair to good yield with excellent regioselectivities under relatively mild reaction conditions. The reaction tolerates a wide range of functional groups. Studies to explore the synthetic potential of this allene oxidation strategy are currently underway.

## Supporting Information

File 1 Experimental procedures and characterization data.

File 2 NMR spectra of compounds.
