# Supplementary material for: Gold-catalyzed regioselective oxidation of terminal allenes: formation of α-methanesulfonyloxy methyl ketones
Source: Beilstein J Org Chem. 2011 May 11;7:596–600. doi: 10.3762/bjoc.7.69 (PMC3107496; doi:10.3762/bjoc.7.69)
Supplement: File 1 — Experimental procedures and characterization data. [file Beilstein_J_Org_Chem-07-596-s001.pdf]

Supporting Information File 1

for

Gold-catalyzed regioselective oxidation of terminal allenes: formation of α-methanesulfonyloxy methyl ketones

Yingdong Luo, Guozhu Zhang, Erik S. Hwang, Thomas A. Wilcoxon and Liming Zhang*

Department of Chemistry and Biochemistry, University of California, Santa Barbara, California, 93106, USA

E-mail: Liming Zhang- zhang@chem.ucsb.edu

* Corresponding author

Experimental procedures and characterization data

Content	Page
**General**	S2
**General procedure A: Preparation of allenes**	S2
**General procedure B: Gold-catalyzed synthesis of α-methanesulfonyloxy methyl ketones**	S3
**^{1}H and ^{13}C NMR spectra (Supporting Information File 2)**	S8

General Ethyl acetate (ACS grade), hexanes (ACS grade), diethyl ether (ACS grade) and anhydrous 1,2-dichloroethane (HPLC grade) were purchased from Fisher Scientific and used without further purification. Methylene chloride and tetrahydrofuran were purified using an MBraun Solvent Purifier. Commercially available reagents were used without further purification. Reactions were monitored by thin layer chromatography (TLC) using Sorbent Technologies' pre-coated silica gel plates. Flash column chromatography was performed over Sorbent Technologies' silica gel (230–400 mesh). ^{1}H NMR and ^{13}C NMR spectra were recorded on a Varian 500 MHz Unity plus spectrometer and a Varian 400 MHz spectrometer using residue solvent peaks as internal standards. Infrared spectra were recorded with a Perkin Elmer FT-IR spectrum 2000 spectrometer and are reported in reciprocal centimeter (cm^{-1}). Mass spectra were recorded with Micromass QTOF2 Quadrupole/Time-of-Flight Tandem mass spectrometer using electron spray ionization.

General procedure A: Preparation of terminal allenes (1a–1k)

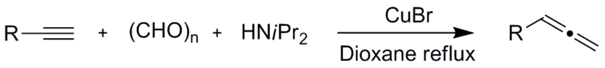

The alkyne (0.01 mol), 20 mL of dioxane, CuBr (0.724 g, 0.00504 mol), paraformaldehyde (0.74 g) and diisopropylamine (1.854 g, 0.0183 mol) were placed in a 50-mL, three-necked flask, equipped with a thermometer, stirrer, and a reflux condenser fitted with a drying tube. The reaction mixture was heated under gentle reflux for 2 h with stirring, then cooled to room temperature and filtered through a Celite plug. The dark-brown filtrate was concentrated under vacuum to afford a gummy residue which was diluted with 5 mL of water followed by 10 mL of ether and acidified with 6 N hydrochloric acid to pH = 2. The ether–water layers were decanted from any residue, the ether layer was separated, and the aqueous solution extracted with ether (5 × 5 mL). The ethereal extracts were combined and washed with small portions of water until the pH was 6.5. The organic layer was then washed with saturated sodium chloride solution and dried over anhydrous $MgSO_4$. After removal of ether by distillation, the residue was distilled in a Kugelrohr apparatus.

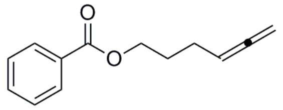

1c

Compound **1c** was prepared in 78% yield according to general procedure A. ^{1}H NMR (400 MHz, $CDCl_3$) δ 8.05 (d, 2H, *J* = 8.0 Hz), 7.55 (t, 1H, *J* = 8.0 Hz), 7.44 (t, 2H, *J* = 7.6 Hz), 5.15–5.08 (m, 1H), 4.70–4.66 (m, 2H), 4.33 (t, 2H, *J* = 6.4 Hz), 2.12–2.05 (m, 2H), 1.86–1.76 (m, 2H), 1.62–1.55 (m, 2H); ^{13}C NMR (100 MHz, $CDCl_3$) δ 208.5, 166.6, 132.8, 130.4, 129.5, 128.3, 89.5, 74.9, 64.8, 28.1, 27.4, 25.4. IR (neat): 3062, 3033, 2941, 2860, 1955, 1719, 1602, 1452, 1314, 1275, 1176, 1116, 1070, 1027, 845; MS (ES^+) Calculated for $[C_{14}H_{16}NaO_2]^+$: 239.10, found: 239.08.

Procedure for preparation of internal allene [1]

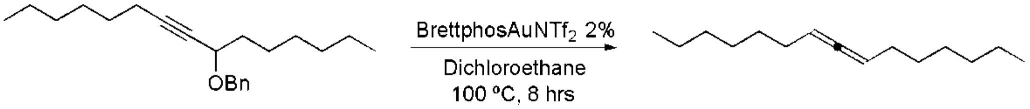

(1-Hexylnon-2-ynyloxymethyl)benzene (314 mg, 1 mmol), BrettPhosAuNTf_2 [2] (20.4 mg, 0.02 mmol) and 2 mL 1,2-dichloroethane were added into a 5 mL vial with a stirring bar. The reaction solution mixture was stirred at 100 °C for 8 h. The reaction mixture was concentrated on a rotary evaporator. The residue was purified by silica gel flash chromatography (eluents: hexanes). Pentadeca-7,8-diene was collected as a colorless oil in 65% yield. ^{1}H NMR (400 MHz, $CDCl_3$) δ 5.06 (quint, 2H, *J* = 4.9 Hz), 2.00–1.94 (m, 4H), 1.41–1.23 (m, 16H), 0.89 (t, 3H, *J* = 6.8 Hz); ^{13}C NMR (100 MHz, $CDCl_3$) δ 203.7, 90.9, 31.7, 29.2, 29.0, 28.8, 22.7, 14.1. IR (neat): 2960, 2856, 1629, 1024, 670; MS (FI) Calculated for $[C_{15}H_{28}]^+$: 208.22, found: 208.22.

General procedure B: preparation of α-methanesulfonyloxy ketones

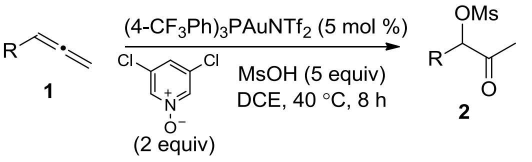

Methanesulfonic acid (1 mmol) and the allene (0.2 mmol) in 2.5 mL of dichloroethane, and (4-$CF_3Ph)_3PAuNTf_2$ (9.5 mg, 0.01 mmol) were added to a solution of 3,5-dichloropyridine *N*-oxide (0.4 mmol). The reaction mixture was stirred at 40 °C for 8 h. Aqueous NH_4Cl solution (2 mL) was added and the mixture extracted with ether (3 × 3 mL). The combined organic layers were concentrated on a rotary evaporator. The residue was purified by silica gel flash chromatography (eluents: first with hexanes / ethyl acetate = 10:1, then with methylene chloride / acetone = 25:1).

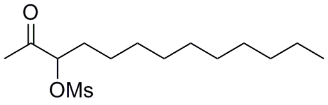

2a

Compound **2a** was prepared in 77% yield according to general procedure B. ^{1}H NMR (500 MHz, $CDCl_3$) δ 4.93 (dd, 1H, *J* = 8.0 Hz, 4.0 Hz), 3.12 (s, 3H), 2.24 (s, 3H), 1.86–1.78 (m, 2H), 1.44–1.40 (m, 2H), 1.34–1.25 (m, 14H), 0.84 (t, 3H, *J* = 6.8); ^{13}C NMR (100 MHz, $CDCl_3$) δ 204.3, 84.2, 38.8, 31.9, 31.2, 29.5, 29.4, 29.3, 29.2, 29.0, 26.2, 24.8, 22.6, 14.1. IR (neat): 2925, 2855, 1733, 1465, 1359, 1177, 953; MS (ES^+) Calculated for $[C_{14}H_{28}NaO_4S]^+$: 315.16, found: 315.10.

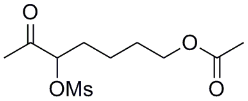

2b

Compound **2b** was prepared in 79% yield according to general procedure B. ^{1}H NMR (500 MHz, $CDCl_3$) δ 4.94 (dd, 1H, *J* = 9.0 Hz, 4.5 Hz), 4.06 (t, 2H, *J* = 6.5 Hz), 3.13 (s, 3H), 2.25 (s, 3H), 2.04 (s, 3H), 1.94 – 1.80 (m, 2H), 1.72 – 1.61 (m, 2H), 1.56 – 1.45 (m, 2H); ^{13}C NMR (100 MHz, $CDCl_3$) δ 204.0, 171.1, 83.7, 63.7, 38.8, 30.8, 28.0, 26.2, 21.4, 21.0. IR (neat): 2942, 1732, 1362, 1245, 1175, 955; MS (ES^+) Calculated for $[C_{10}H_{18}NaO_6S]^+$: 289.07, found: 289.09.

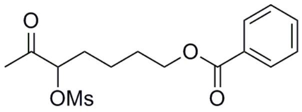

2c

Compound **2c** was prepared in 75% yield according to general procedure B. ^{1}H NMR (500 MHz, $CDCl_3$) δ 8.03 (d, 2H, *J* = 8.0 Hz), 7.56 (t, 1H, *J* = 7.2 Hz), 7.44 (t, 2H, *J* = 7.5 Hz), 4.97 (dd, 1H, *J* = 8.0 Hz, 4.0 Hz), 4.34 (t, 2H, *J* = 6.3 Hz), 3.12 (s, 3H), 2.26 (s, 3H), 2.00–1.78 (m, 4H), 1.65–1.59 (m, 2H); ^{13}C NMR (100 MHz, $CDCl_3$) δ 204.0, 166.6, 133.0, 130.1, 129.5, 128.4,

85.7, 64.2, 38.8, 30.8, 28.1, 26.2, 21.5. IR (neat): 2942, 1720, 1601, 1451, 1359, 1276, 1175, 1071, 917; MS (ES^+) Calculated for $[C_{15}H_{20}NaO_6S]^+$: 351.09, found: 351.10.

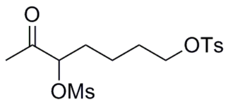

2d

Compound **2d** was prepared in 80% yield according to general procedure B. ^{1}H NMR (400 MHz, $CDCl_3$) δ 7.77 (d, 2H, *J* = 8.4 Hz), 7.35 (d, 2H, *J* = 8.4 Hz), 4.89 (dd, 1H, *J* = 8.3 Hz, 4.1 Hz), 4.02 (t, 2H, *J* = 6.2 Hz), 3.12 (s, 3H), 2.44 (s, 3H), 2.23 (s, 3H), 1.88–1.63 (m, 4H), 1.52–1.44 (m, 2H); ^{13}C NMR (100 MHz, $CDCl_3$) δ 203.9, 144.9, 132.7, 129.9, 127.8, 83.4, 69.7, 38.8, 30.4, 28.1, 26.2, 21.6, 20.9. IR (neat): 2940, 1771, 1710, 1437, 1399, 1360, 1176, 1043, 955, 720; MS (ES^+) Calculated for $[C_{15}H_{22}NaO_7S_2]^+$: 401.07, found: 401.09.

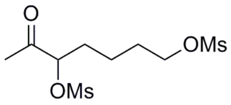

2e

Compound **2e** was prepared in 73% yield according to general procedure B. ^{1}H NMR (500 MHz, $CDCl_3$) δ 4.94 (dd, 1H, *J* = 8.6 Hz, 4.3 Hz), 4.23 (t, 2H, *J* = 6.5 Hz), 3.13 (s, 3H), 3.00 (s, 3H), 2.25 (s, 3H), 1.96–1.73 (m, 4H), 1.60–1.54 (m, 2H); ^{13}C NMR (100 MHz, $CDCl_3$) δ 204.0, 83.4, 69.2, 38.9, 37.4, 30.4, 28.4, 26.3, 20.9. IR (neat): 2940, 1735, 1598, 1355, 1176, 1098, 951; MS (ES^+) Calculated for $[C_9H_{18}NaO_7S_2]^+$: 325.04, found: 325.06.

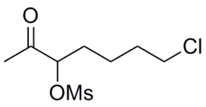

2f

Compound **2f** was prepared in 63% yield according to general procedure B. ^{1}H NMR (500 MHz, $CDCl_3$) δ 4.95 (dd, 1H, *J* = 8.3 Hz, 4.1Hz), 3.55 (t, 2H, *J* = 6.5 Hz), 3.14 (s, 3H), 2.27 (s, 3H), 1.92–1.79 (m, 4H), 1.64–1.58 (m, 2H); ^{13}C NMR (100 MHz, $CDCl_3$) δ 204.0, 83.5, 44.3, 38.9, 31.7, 30.5, 26.3, 22.1. IR (neat): 2942, 1735, 1356, 1174, 948; MS (ES^+) Calculated for $[C_8H_{15}ClNaO_4S]^+$: 265.03, found: 265.05.

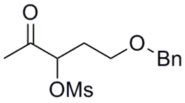

2g

Compound **2g** was prepared in 61% yield according to general procedure B. ^{1}H NMR (500 MHz, $CDCl_3$) δ 7.37– 7.28 (m, 5H), 5.14 (dd, 1H, *J* = 8.0 Hz, 4.0 Hz), 4.48 (s, 2H), 3.66–3.58 (m, 2H), 3.08 (s, 3H), 2.24 (s, 3H), 2.24–2.18 (m, 1H), 2.14–2.06 (m, 1H); ^{13}C NMR (100 MHz, $CDCl_3$) δ 204.0, 137.6, 128.4, 127.8, 81.4, 73.2, 64.5, 38.6, 31.7, 26.2. IR (neat): 3030, 2942, 2868, 1733, 1454, 1419, 1360, 1176, 1094, 951; MS (ES^+) Calculated for $[C_{13}H_{18}NaO_5S]^+$: 309.08, found: 309.10

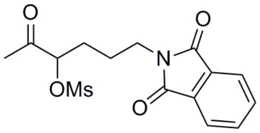

2h

Compound **2h** was prepared in 76% yield according to general procedure B. ^{1}H NMR (500 MHz, $CDCl_3$) δ 7.85–7.84 (m, 2H), 7.74–7.72 (m, 2H), 5.02 (dd, 1H, *J* = 7.7 Hz, 3.8 Hz), 3.74 (t, 2H, *J* = 6.2 Hz), 3.14 (s, 3H), 2.25 (s, 3H), 1.93–1.82 (m, 4H); ^{13}C NMR (100 MHz, $CDCl_3$) δ 203.9, 168.3, 134.1, 132.0, 123.3, 82.9, 38.8, 36.8, 28.3, 26.2, 24.1. IR (neat): 2959, 2872, 1719, 1419, 1358, 1235, 1176, 942, 840; MS (ES^+) Calculated for $[C_{15}H_{17}NNaO_6S]^+$: 362.07, found: 362.09.

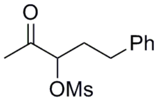

2i

Compound **2i** was prepared in 60% yield according to general procedure B. ^{1}H NMR (500 MHz, $CDCl_3$) δ 7.32 (t, 2H, *J* = 7.5 Hz), 7.24–7.20 (m, 3H), 4.95 (dd, 1H, *J* = 8.3 Hz, 4.1 Hz), 3.12 (s, 3H), 2.84–2.73 (m, 2H), 2.22 (s, 3H), 2.20–2.09 (m, 2H); ^{13}C NMR (100 MHz, $CDCl_3$) δ 204.1, 139.7, 128.9, 128.6, 126.7, 83.4, 39.0, 32.9, 31.1, 26.3. IR (neat): 2937, 1731, 1497, 1354, 1174, 945; MS (ES^+) Calculated for $[C_{12}H_{16}O_4S + H_2O]^+$: 274.09, found: 274.13.

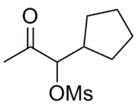

2j

Compound **2j** was prepared in 72% yield according to general procedure B. ^{1}H NMR (500 MHz, $CDCl_3$) δ 4.84 (d, 1H, *J* = 5.5 Hz), 3.11 (s, 3H), 2.40–2.35 (m, 1H), 2.26 (s, 3H), 1.78–1.64 (m, 4H), 1.60–1.54 (m, 2H), 1.50–1.42 (m, 2H); ^{13}C NMR (100 MHz, $CDCl_3$) δ 204.1, 86.6, 40.7, 38.6, 28.6, 27.3, 26.5, 25.3, 25.2. IR (neat): 2942, 2857, 1734, 1419, 1351, 1172, 957; MS (ES^+) Calculated for $[C_9H_{16}NaO_4S]^+$: 243.07, found: 243.08.

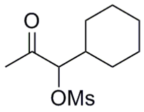

2k

Compound **2k** was prepared in 59% yield according to general procedure B. ^{1}H NMR (500 MHz, $CDCl_3$) δ 4.73 (d, 1H, *J* = 4.5 Hz), 3.10 (s, 3H), 2.24 (s, 3H), 1.96–1.91 (m, 1H), 1.82–1.77 (m, 2H), 1.68–1.64 (m, 2H), 1.58–1.56 (m, 2H), 1.33–1.12 (m, 5H); ^{13}C NMR (100 MHz, $CDCl_3$) δ 204.5, 88.2, 39.7, 38.6, 29.2, 27.1, 26.8, 25.9, 25.7, 25.6. IR (neat): 2932, 2856, 1719, 1452, 1360, 1174, 951, 837; MS (ES^+) Calculated for $[C_{10}H_{18}NaO_4S]^+$: 257.08, found: 257.11.

References

1. This procedure is based on the work of Gagosz (Bolte, B.; Odabachian, Y.; Gagosz, F. *J. Am. Chem. Soc.* **2010**, *132*, 7294–7296) with the exception that a different gold catalyst was used.
2. For the synthesis and structure of this gold complex, see: Ye, L.; He, W.; Zhang, L. *Angew. Chem., Int. Ed.* **2011**, *50*, 3236–3239. For Buchwald's initial report of BrettPhos, a bulky biaryl-based phosphine ligand, see: Fors, B. P.; Watson, D. A.; Biscoe, M. R.; Buchwald, S. L. *J. Am. Chem. Soc.* **2008**, *130*, 13552–13554.
