# Supplementary material for: Gold-catalyzed regioselective oxidation of terminal allenes: formation of α-methanesulfonyloxy methyl ketones
Source: Beilstein J Org Chem. 2011 May 11;7:596–600. doi: 10.3762/bjoc.7.69 (PMC3107496; doi:10.3762/bjoc.7.69)

Supporting Information File 2

for

Gold-catalyzed regioselective oxidation of terminal allenes: formation of α-methanesulfonyloxy methyl ketones

Yingdong Luo, Guozhu Zhang, Erik S. Hwang, Thomas A. Wilcoxon and Liming Zhang*

Address: Department of Chemistry and Biochemistry, University of California, Santa Barbara, California, 93106, USA

Email:Liming Zhang- zhang@chem.ucsb.edu

* Corresponding author

NMR spectra of compounds

lyd-1-212-4

File: nmr400/Zhang/yluo/lyd-1-212-4.fid

Pulse Sequence: s2pul

Solvent: cdcl3
Ambient temperature
Operator: yluo
File: lyd-1-212-4
INOVA-500 "nmrserver"

Relax. delay 4.800 sec
Pulse 75.0 degrees
Acq. time 2.500 sec
Width 8002.4 Hz
36 repetitions
OBSERVE H1, 399.9486723 MHz
DATA PROCESSING
Line broadening 0.2 Hz
FT size 65536
Total time 14 min, 39 sec

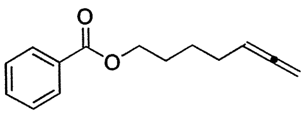

1c

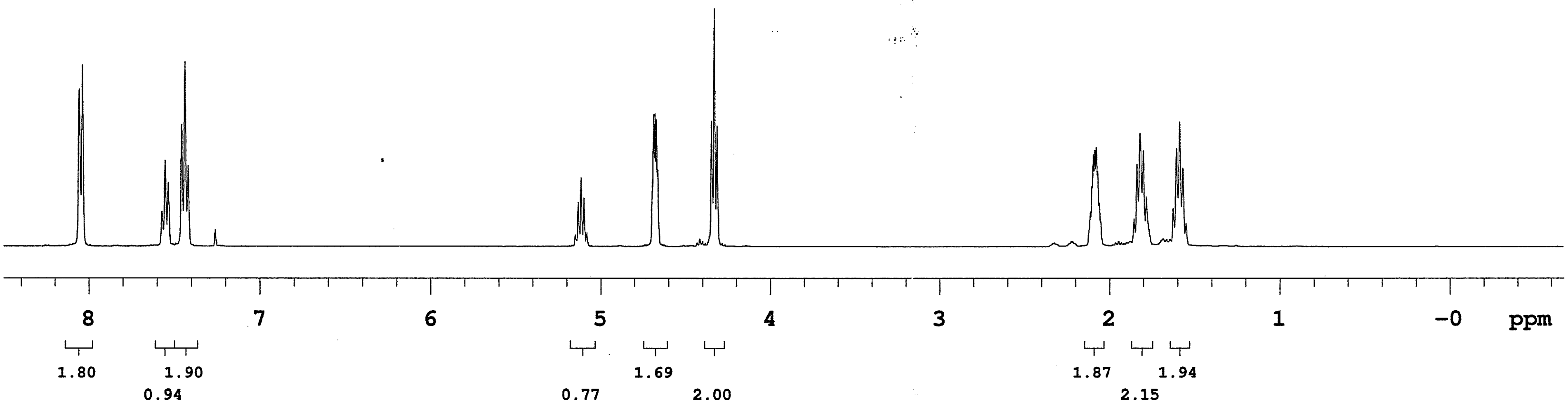

lyd-1-212-4-c

File: nmr400/Zhang/yluo/lyd-1-212-4-c.fid

Pulse Sequence: s2pul

Solvent: cdcl3
Ambient temperature
Operator: yluo
File: lyd-1-212-4-c
INOVA-500 "nmrserver"

Relax. delay 1.300 sec
Pulse 53.8 degrees
Acq. time 1.000 sec
Width 28040.7 Hz
116 repetitions
OBSERVE C13, 100.5671787 MHz
DECOUPLE H1, 399.9506784 MHz
Power 40 dB
continuously on
WALTZ-16 modulated
DATA PROCESSING
Line broadening 1.0 Hz
FT size 65536
Total time 6 hr, 24 min, 59 sec

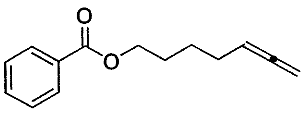

1c

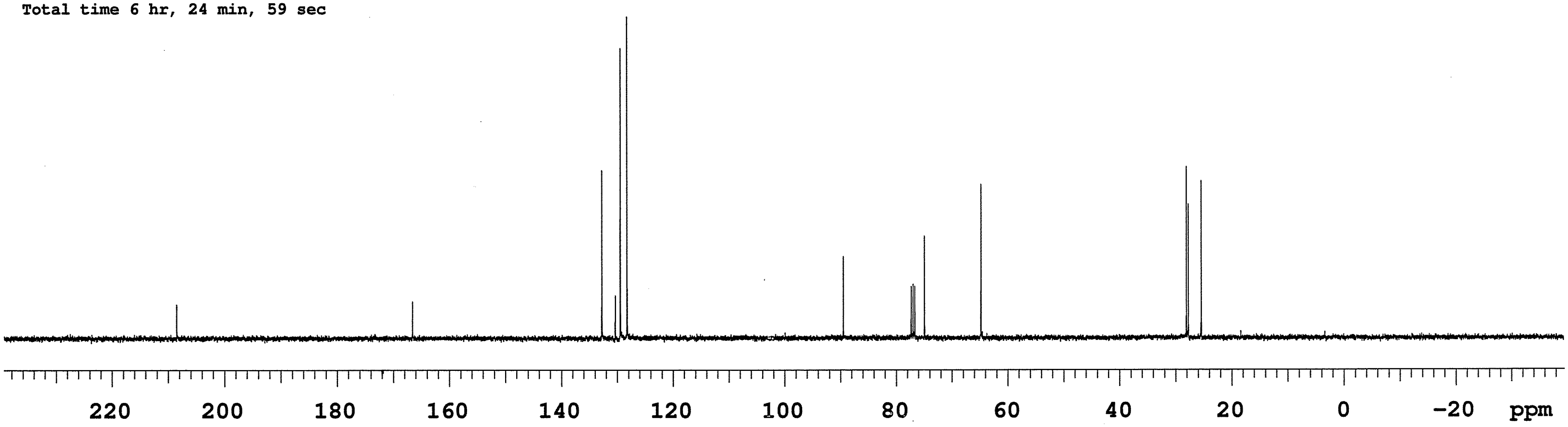

lyd-1-212-C12

File: xp

Pulse Sequence: s2pul
 Solvent: cdcl3
 Ambient temperature
Operator: yluo
INOVA-500 "nmr500"

 Relax. delay 4.800 sec
 Pulse 74.9 degrees
 Acq. time 2.500 sec
 Width 8002.4 Hz
 12 repetitions
OBSERVE H1, 499.8560510 MHz
DATA PROCESSING
 Line broadening 0.2 Hz
FT size 32768
Total time 14 min, 37 sec

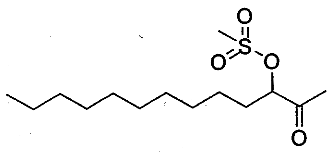

2a

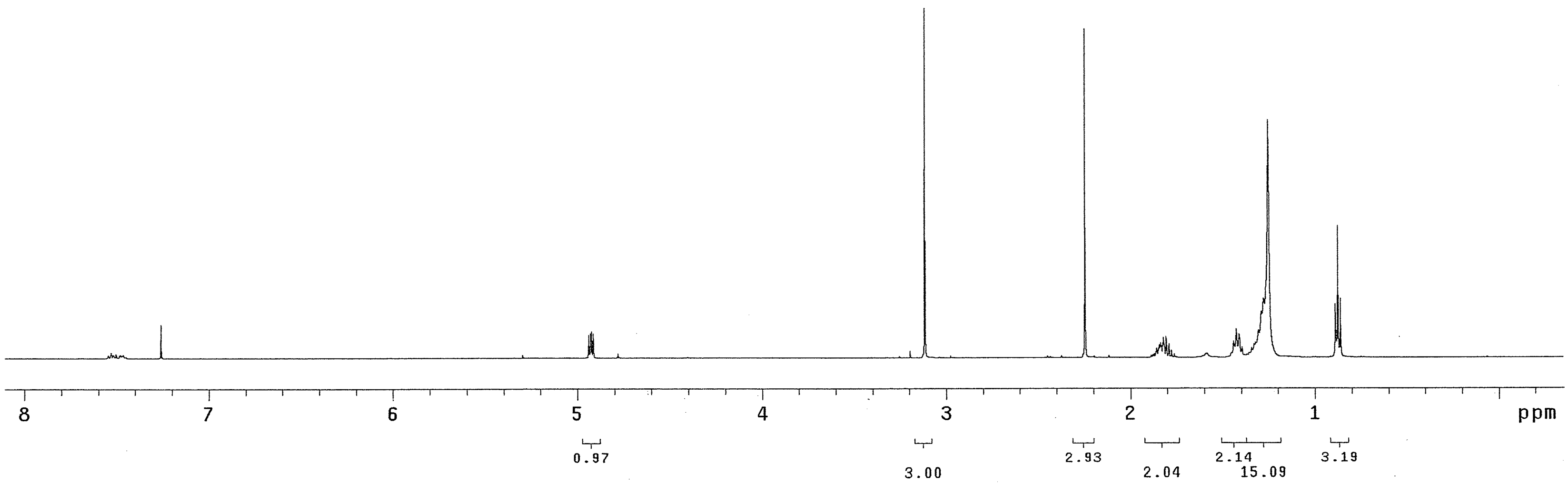

lyd-1-212-C12-c

File: xp

Pulse Sequence: s2pul

Solvent: cdcl3
Ambient temperature
Operator: yluo
INOVA-400 "nmr400"

Relax. delay 1.300 sec
Pulse 53.8 degrees
Acq. time 1.000 sec
Width 28040.7 Hz
188 repetitions
OBSERVE C13, 100.5671761 MHz
DECOUPLE H1, 399.9506784 MHz
Power 40 dB
continuously on
WALTZ-16 modulated
DATA PROCESSING
Line broadening 1.0 Hz
FT size 65536
Total time 7 hr, 41 min, 56 sec

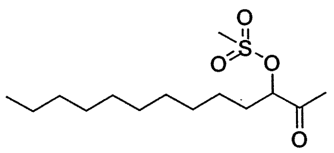

2a

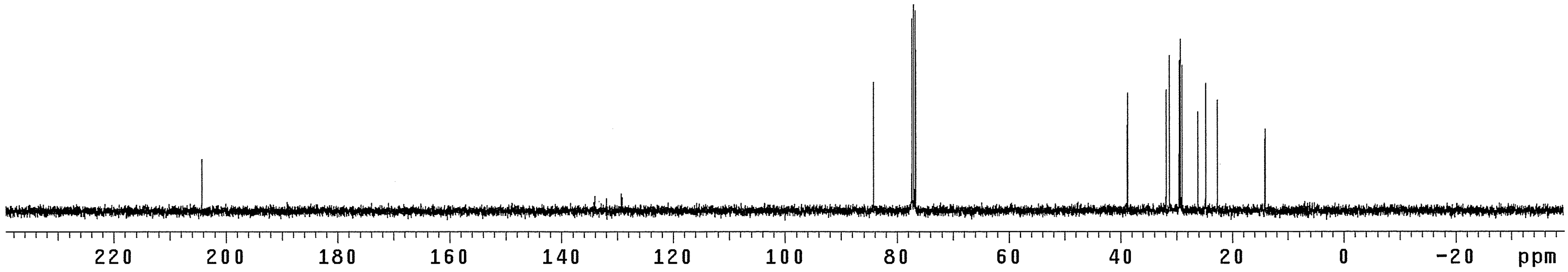

lyd-1-204

File: nmr500/Zhang/yluo/lyd-1-204.fid

Pulse Sequence: s2pul

Solvent: cdcl3
Ambient temperature
Operator: yluo
File: lyd-1-204
INOVA-500 "nmrserver"

Relax. delay 4.800 sec
Pulse 74.9 degrees
Acq. time 2.500 sec
Width 8002.4 Hz
8 repetitions
OBSERVE H1, 499.8560510 MHz
DATA PROCESSING
Line broadening 0.2 Hz
FT size 32768
Total time 0 min, 0 sec

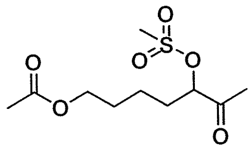

2b

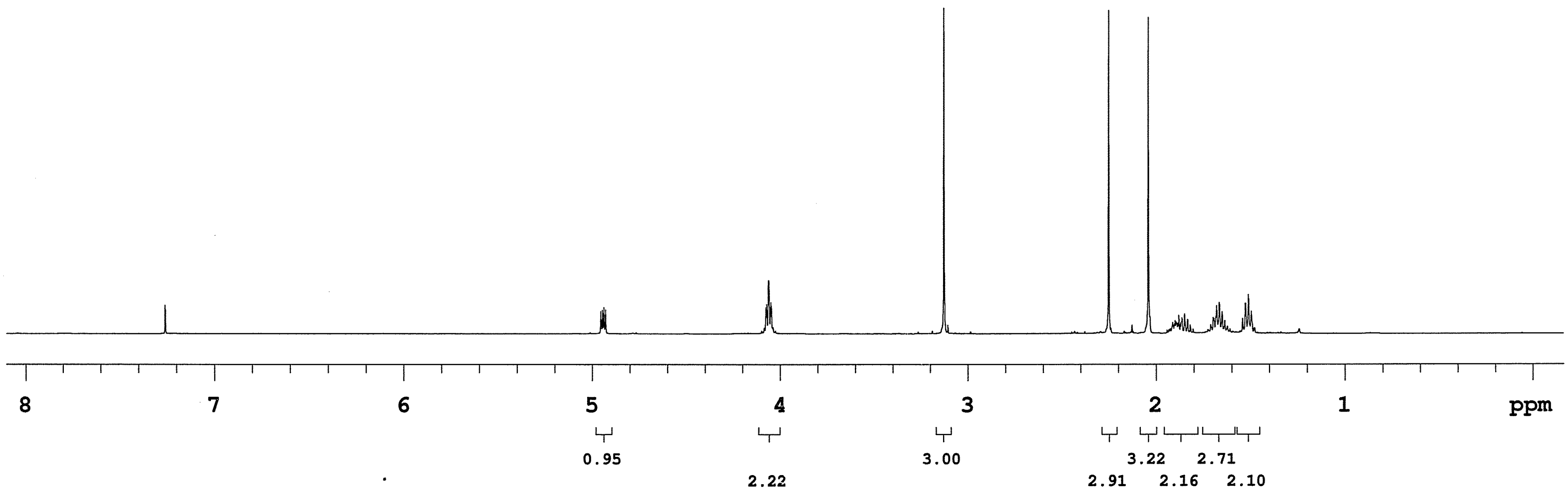

lyd-1-204-c

File: nmr400/Zhang/yluo/lyd-1-204-c.fid

Pulse Sequence: s2pul

Solvent: cdcl3
Ambient temperature
Operator: yluo
File: lyd-1-204-c
INOVA-500 "nmrserver"

Relax. delay 1.300 sec
Pulse 53.8 degrees
Acq. time 1.000 sec
Width 28040.7 Hz
1012 repetitions
OBSERVE C13, 100.5671770 MHz
DECOUPLE H1, 399.9506784 MHz
Power 40 dB
continuously on
WALTZ-16 modulated
DATA PROCESSING
Line broadening 1.0 Hz
FT size 65536
Total time 6 hr, 24 min, 59 sec

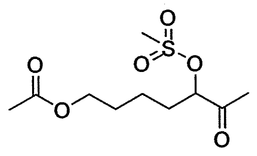

2b

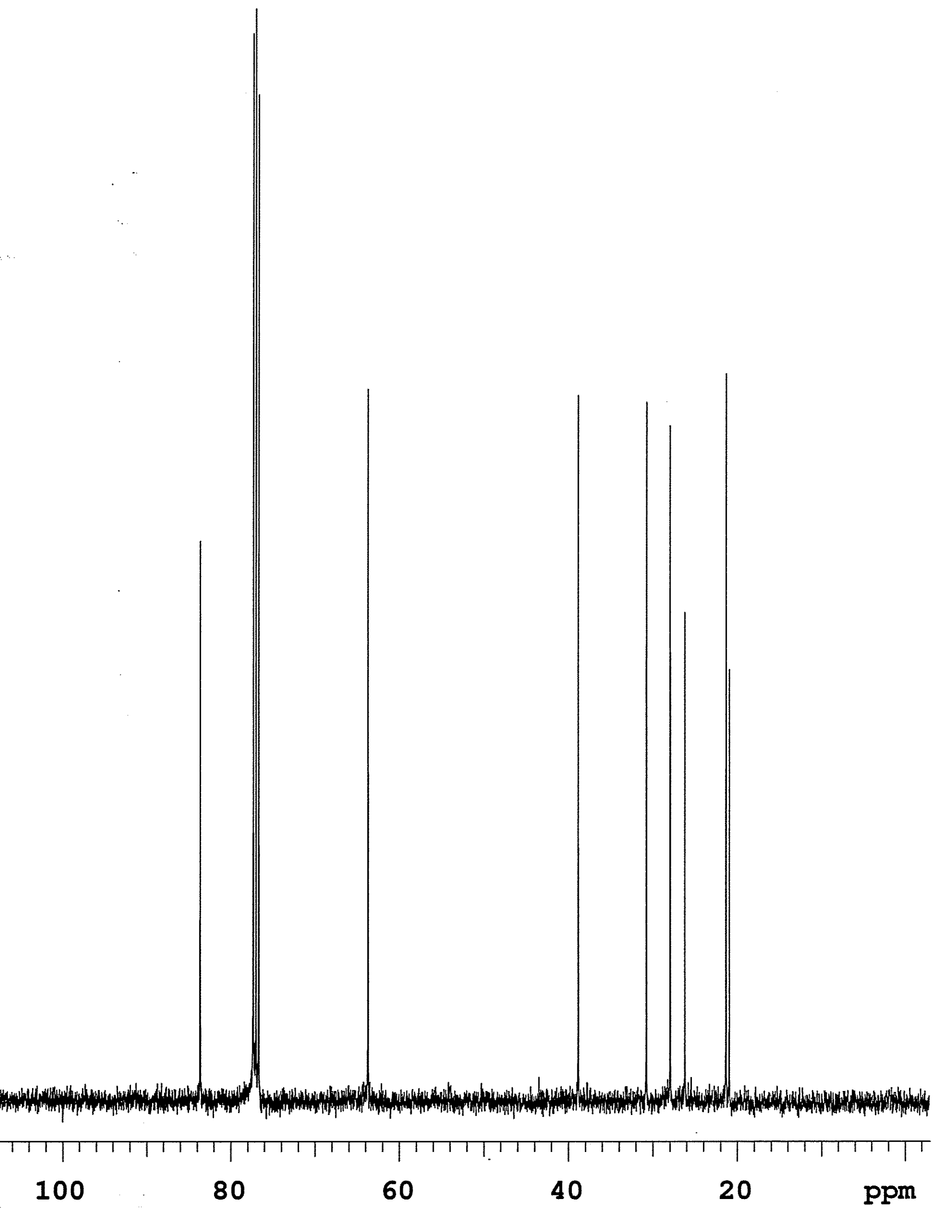

lyd-1-206-1

File: nmr500/Zhang/yluo/lyd-1-206-1.fid

Pulse Sequence: s2pul

Solvent: cdcl3
Ambient temperature
Operator: yluo
File: lyd-1-206-1
INOVA-500 "nmrserver"

Relax. delay 4.800 sec
Pulse 74.9 degrees
Acq. time 2.500 sec
Width 8002.4 Hz
12 repetitions
OBSERVE H1, 499.8560510 MHz
DATA PROCESSING
Line broadening 0.2 Hz
FT size 32768
Total time 0 min, 0 sec

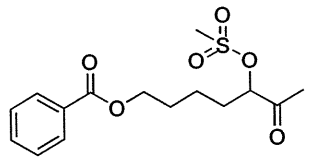

2c

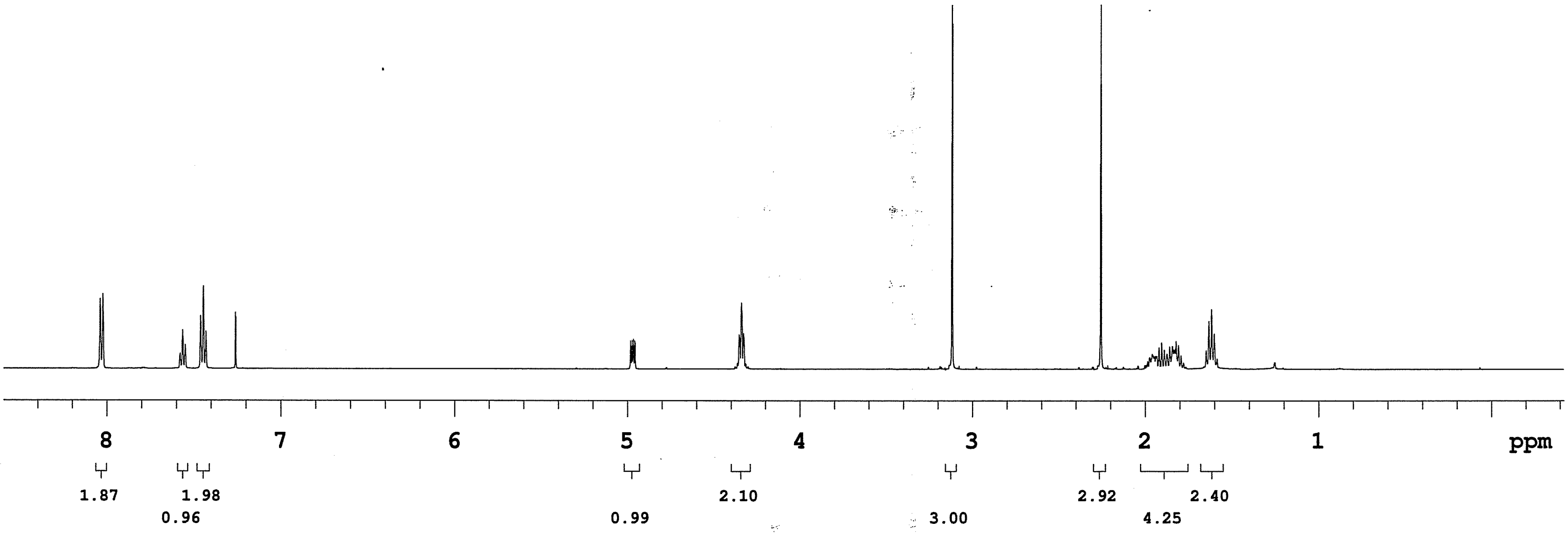

lyd-1-206-1-c

File: nmr400/Zhang/yluo/lyd-1-206-1-c.fid

Pulse Sequence: s2pul

Solvent: cdcl3
Ambient temperature
Operator: yluo
File: lyd-1-206-1-c
INOVA-500 "nmrserver"

Relax. delay 1.300 sec
Pulse 53.8 degrees
Acq. time 1.000 sec
Width 28040.7 Hz
1024 repetitions
OBSERVE C13, 100.5671761 MHz
DECOUPLE H1, 399.9506784 MHz
Power 40 dB
continuously on
WALTZ-16 modulated
DATA PROCESSING
Line broadening 1.0 Hz
FT size 65536
Total time 7 hr, 41 min, 56 sec

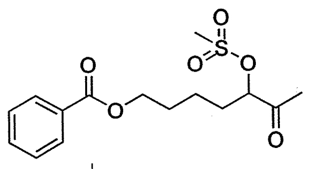

2c

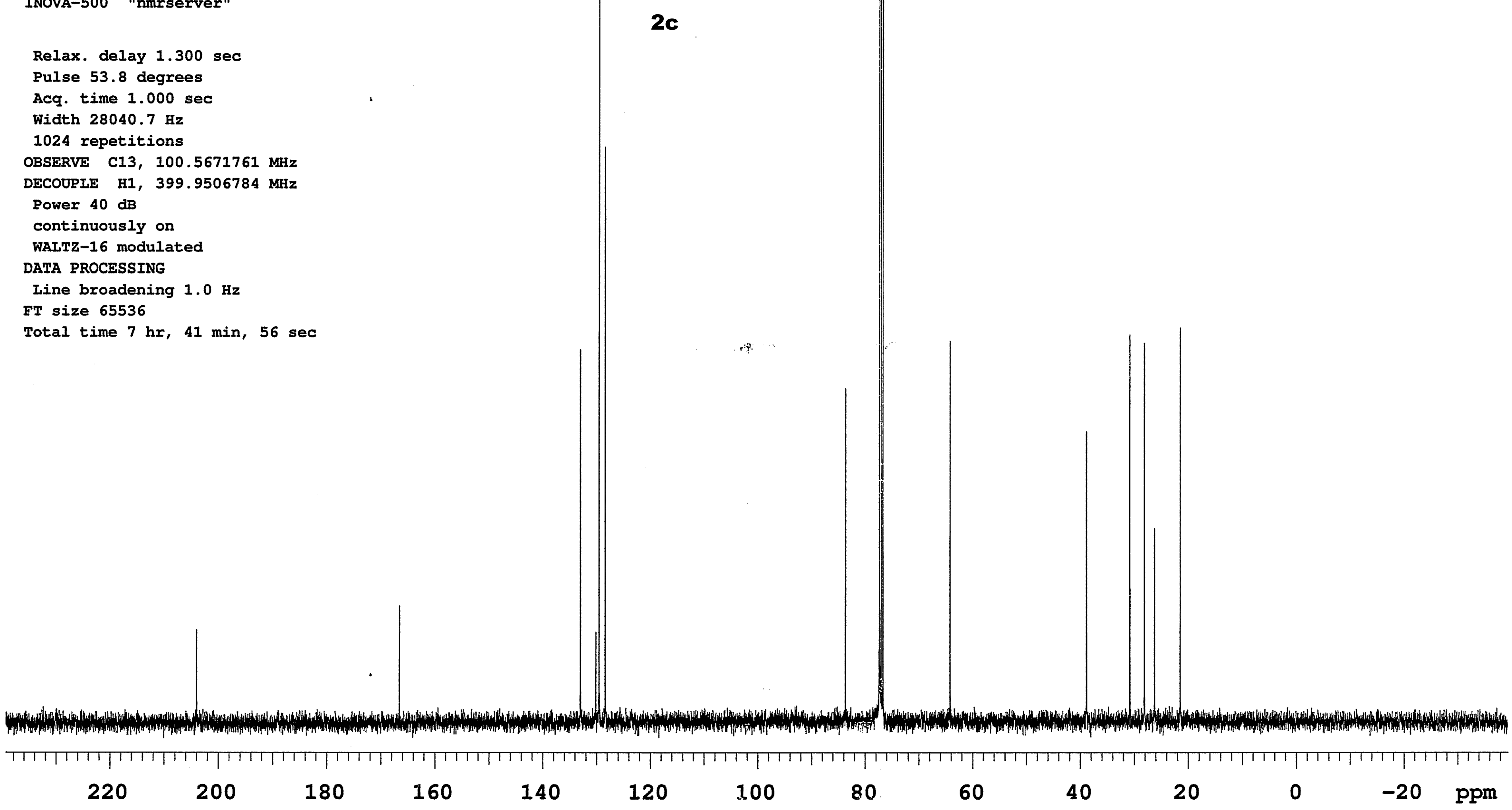

lyd-1-212-1

File: nmr400/Zhang/yluo/lyd-1-212-1.fid

Pulse Sequence: s2pul

Solvent: cdcl3
Ambient temperature
Operator: yluo
File: lyd-1-212-1
INOVA-500 "nmrserver"

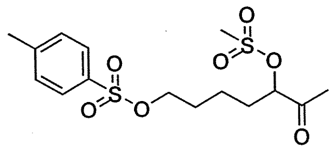

2d

Relax. delay 4.800 sec
Pulse 75.0 degrees
Acq. time 2.500 sec
Width 8002.4 Hz
8 repetitions
OBSERVE H1, 399.9486723 MHz
DATA PROCESSING
Line broadening 0.2 Hz
FT size 65536
Total time 14 min, 39 sec

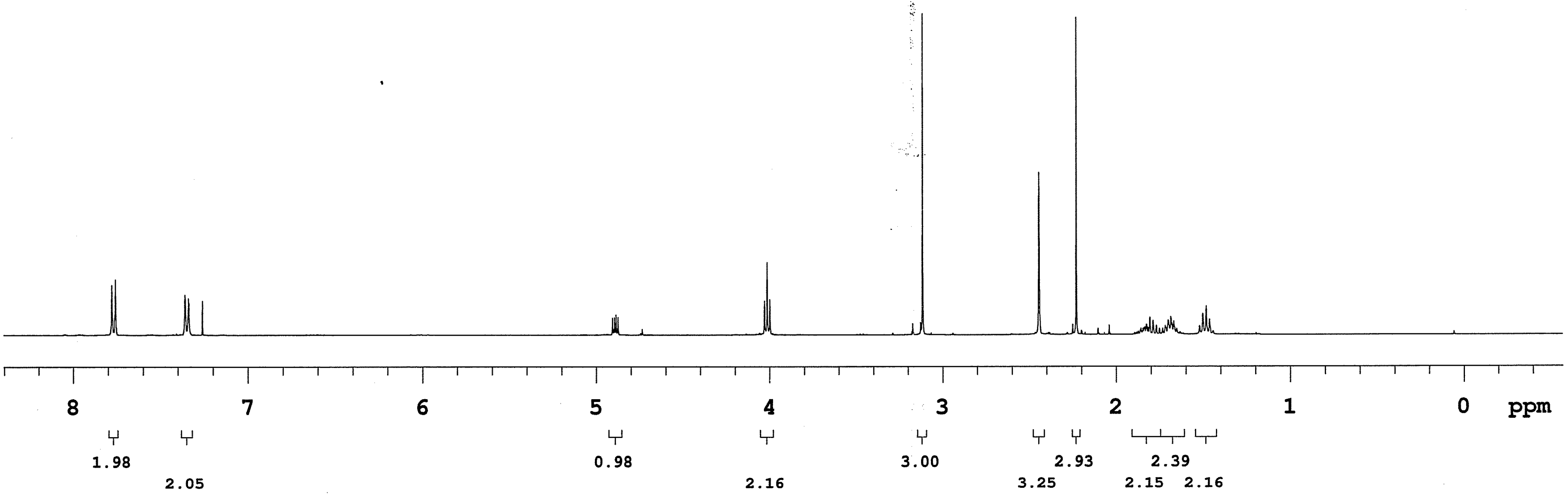

lyd-1-212-1-c

File: nmr400/Zhang/yluo/lyd-1-212-1-c.fid

Pulse Sequence: s2pul

Solvent: cdcl3
Ambient temperature
Operator: yluo
File: lyd-1-212-1-c
INOVA-500 "nmrserver"

Relax. delay 1.300 sec
Pulse 53.8 degrees
Acq. time 1.000 sec
Width 28040.7 Hz
52 repetitions
OBSERVE C13, 100.5671796 MHz
DECOUPLE H1, 399.9506784 MHz
Power 40 dB
continuously on
WALTZ-16 modulated
DATA PROCESSING
Line broadening 1.0 Hz
FT size 65536
Total time 7 hr, 41 min, 56 sec

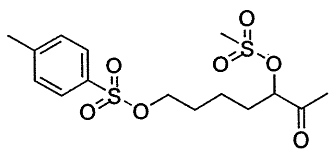

2d

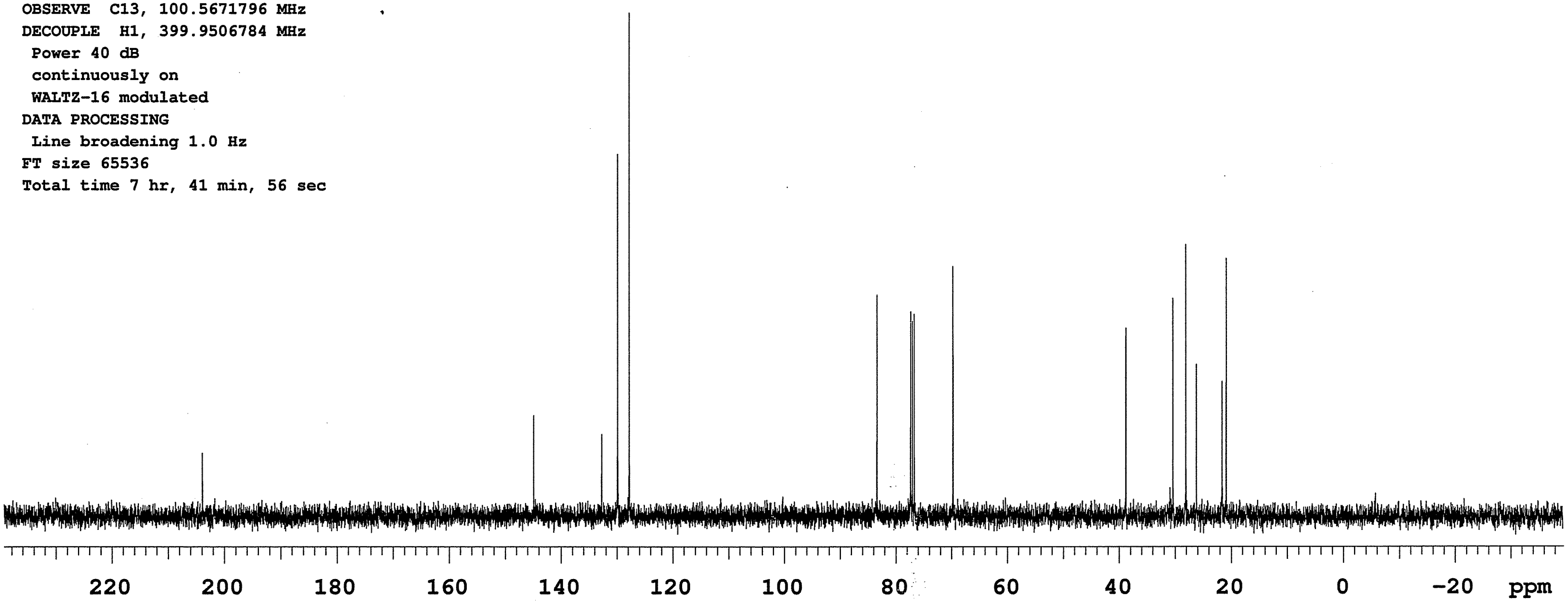

Standard c13 run using qnp probe

File: nmr400/Zhang/yluo/lyd-1-208-4.fid

Pulse Sequence: s2pul

Solvent: cdcl3
Ambient temperature
Operator: yluo
File: lyd-1-208-4
INOVA-500 "nmrserver"

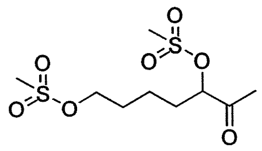

2e

Relax. delay 1.300 sec
Pulse 53.8 degrees
Acq. time 1.000 sec
Width 28040.7 Hz
1312 repetitions
OBSERVE C13, 100.5671752 MHz
DECOUPLE H1, 399.9506784 MHz
Power 40 dB
continuously on
WALTZ-16 modulated
DATA PROCESSING
Line broadening 1.0 Hz
FT size 65536
Total time 6 hr, 24 min, 59 sec

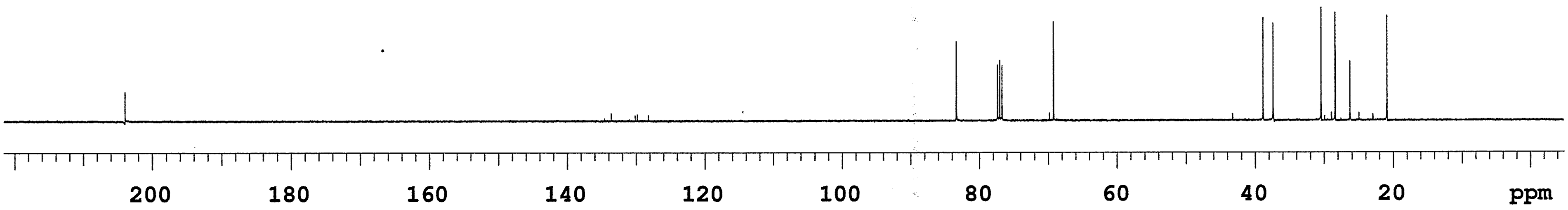

lyd-1-208-4

File: nmr500/Zhang/yluo/lyd-1-208-4.fid

Pulse Sequence: s2pul

Solvent: cdcl3
Ambient temperature
Operator: yluo
File: lyd-1-208-4
INOVA-500 "nmrserver"

Relax. delay 4.800 sec
Pulse 74.9 degrees
Acq. time 2.500 sec
Width 8002.4 Hz
16 repetitions
OBSERVE H1, 499.8560510 MHz
DATA PROCESSING
Line broadening 0.2 Hz
FT size 32768
Total time 0 min, 0 sec

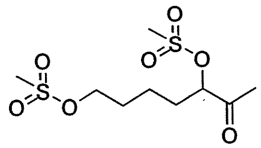

2e

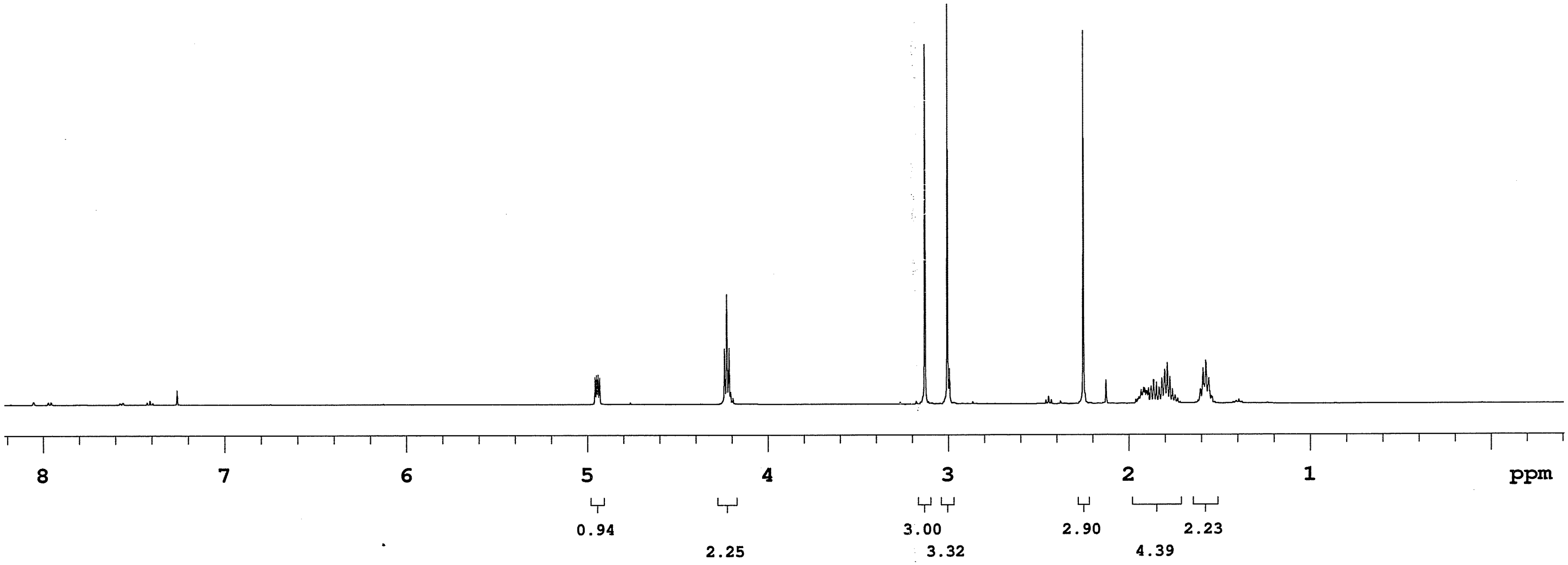

lyd-1-206-2

File: nmr500/Zhang/yluo/lyd-1-206-2.fid

Pulse Sequence: s2pul

Solvent: cdcl3
Ambient temperature
Operator: yluo
File: lyd-1-206-2
INOVA-500 "nmrserver"

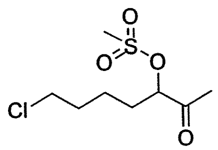

2f

Relax. delay 4.800 sec
Pulse 74.9 degrees
Acq. time 2.500 sec
Width 8002.4 Hz
12 repetitions
OBSERVE H1, 499.8560510 MHz
DATA PROCESSING
Line broadening 0.2 Hz
FT size 32768
Total time 0 min, 0 sec

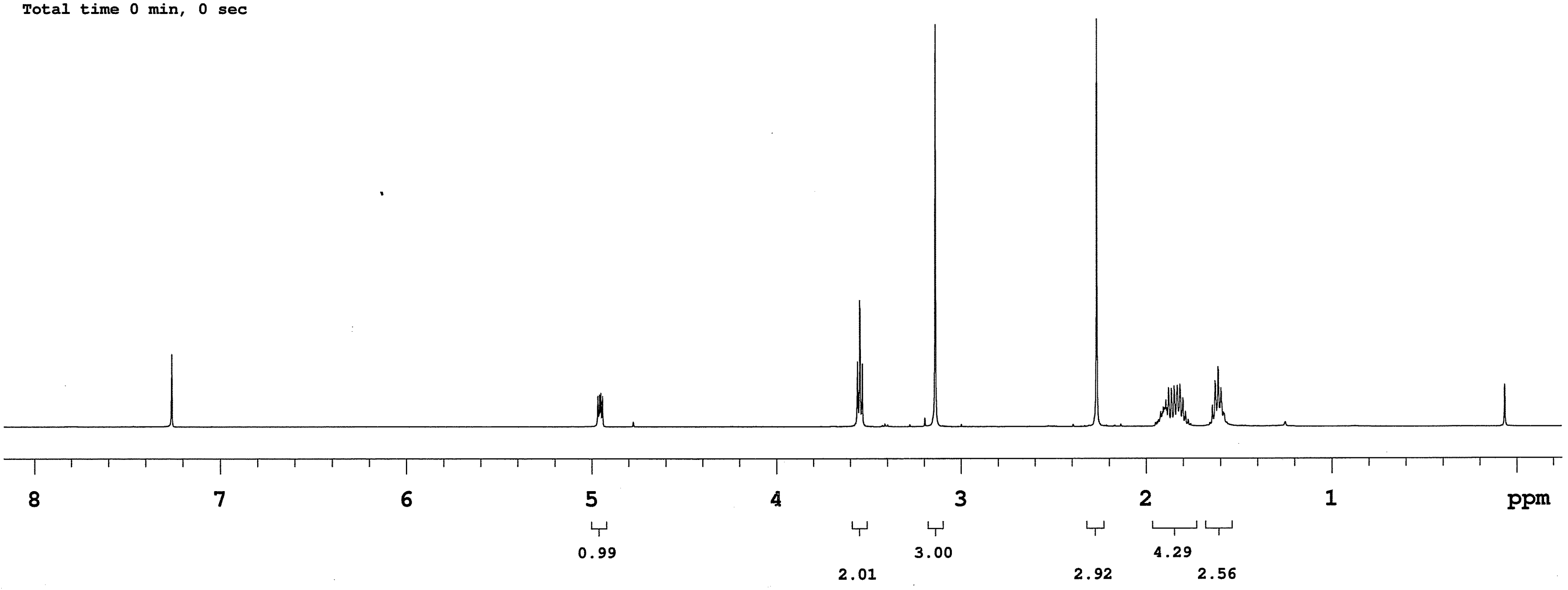

lyd-1-206-2-c

File: nmr400/Zhang/yluo/lyd-1-206-2-c.fid

Pulse Sequence: s2pul

Solvent: cdcl3
Ambient temperature
Operator: yluo
File: lyd-1-206-2-c
INOVA-500 "nmrserver"

Relax. delay 1.300 sec
Pulse 53.8 degrees
Acq. time 1.000 sec
Width 28040.7 Hz
1028 repetitions
OBSERVE C13, 100.5671761 MHz
DECOUPLE H1, 399.9506784 MHz
Power 40 dB
continuously on
WALTZ-16 modulated
DATA PROCESSING
Line broadening 1.0 Hz
FT size 65536
Total time 46 min, 21 sec

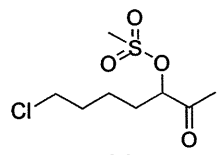

2f

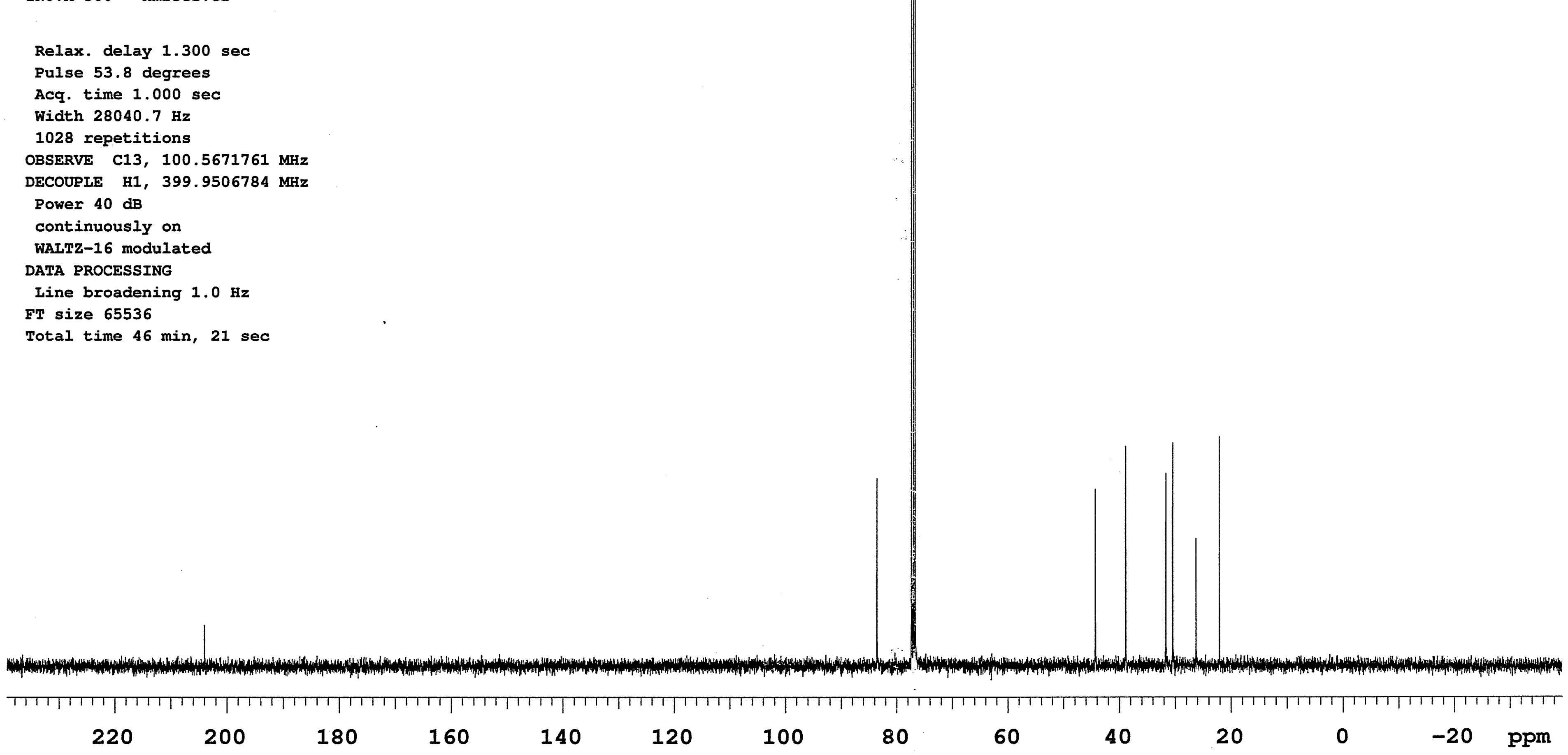

lyd-1-206-3

File: nmr500/Zhang/yluo/lyd-1-206-3.fid

Pulse Sequence: s2pul

Solvent: cdcl3
Ambient temperature
Operator: yluo
File: lyd-1-206-3
INOVA-500 "nmrserver"

Relax. delay 4.800 sec
Pulse 74.9 degrees
Acq. time 2.500 sec
Width 8002.4 Hz
16 repetitions
OBSERVE H1, 499.8560510 MHz
DATA PROCESSING
Line broadening 0.2 Hz
FT size 32768
Total time 0 min, 0 sec

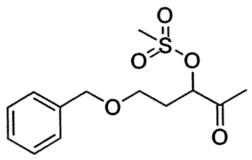

2g

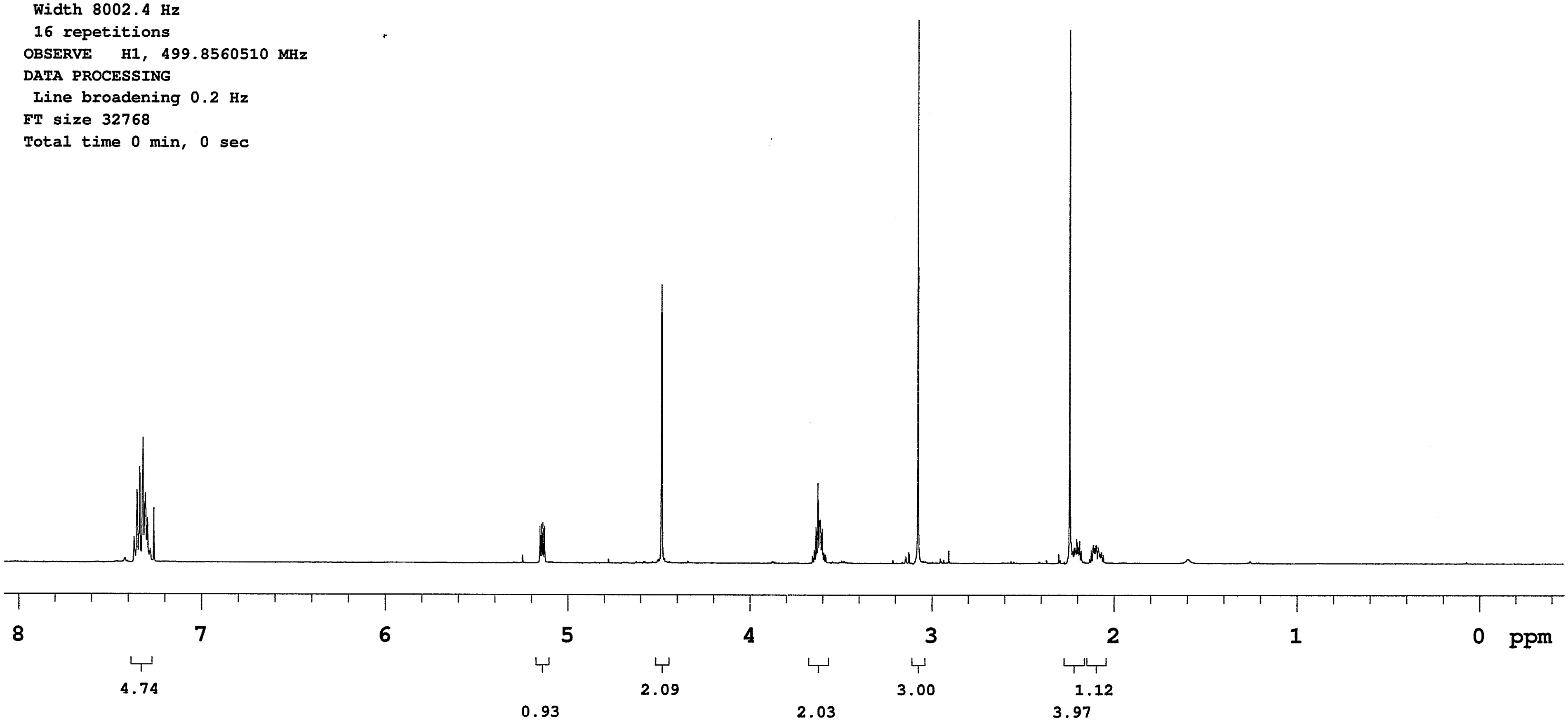

lyd-1-206-3-c

File: nmr400/Zhang/yluo/lyd-1-206-3-c.fid

Pulse Sequence: s2pul

Solvent: cdcl3
Ambient temperature
Operator: yluo
File: lyd-1-206-3-c
INOVA-500 "nmrserver"

Relax. delay 1.300 sec
Pulse 53.8 degrees
Acq. time 1.000 sec
Width 28040.7 Hz
756 repetitions
OBSERVE C13, 100.5671770 MHz
DECOUPLE H1, 399.9506784 MHz
Power 40 dB
continuously on
WALTZ-16 modulated
DATA PROCESSING
Line broadening 1.0 Hz
FT size 65536
Total time 7 hr, 41 min, 56 sec

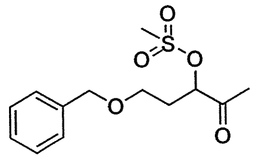

2g

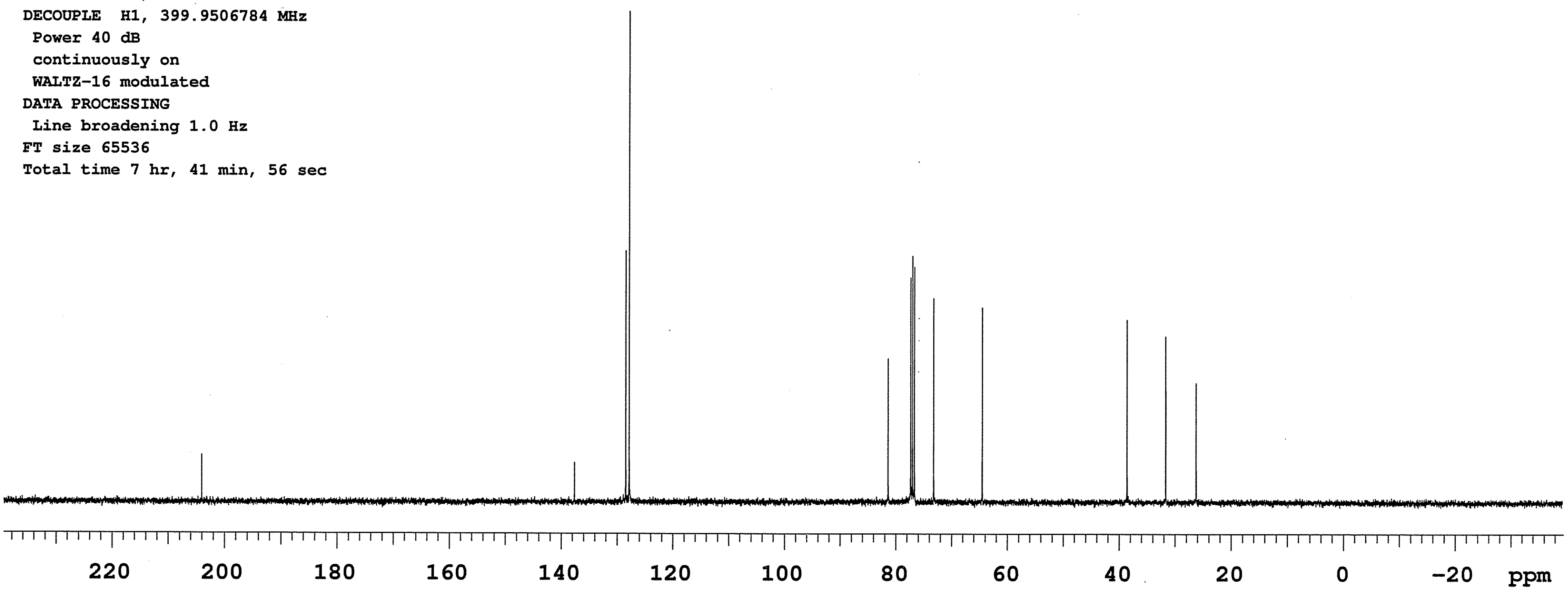

lyd-1-208-1

File: nmr500/Zhang/yluo/lyd-1-208-1.fid

Pulse Sequence: s2pul

Solvent: cdcl3
Ambient temperature
Operator: yluo
File: lyd-1-208-1
INOVA-500 "nmrserver"

Relax. delay 4.800 sec
Pulse 74.9 degrees
Acq. time 2.500 sec
Width 8002.4 Hz
12 repetitions
OBSERVE H1, 499.8560510 MHz
DATA PROCESSING
Line broadening 0.2 Hz
FT size 32768
Total time 0 min, 0 sec

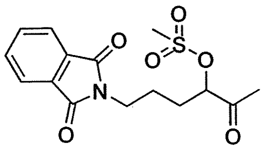

2h

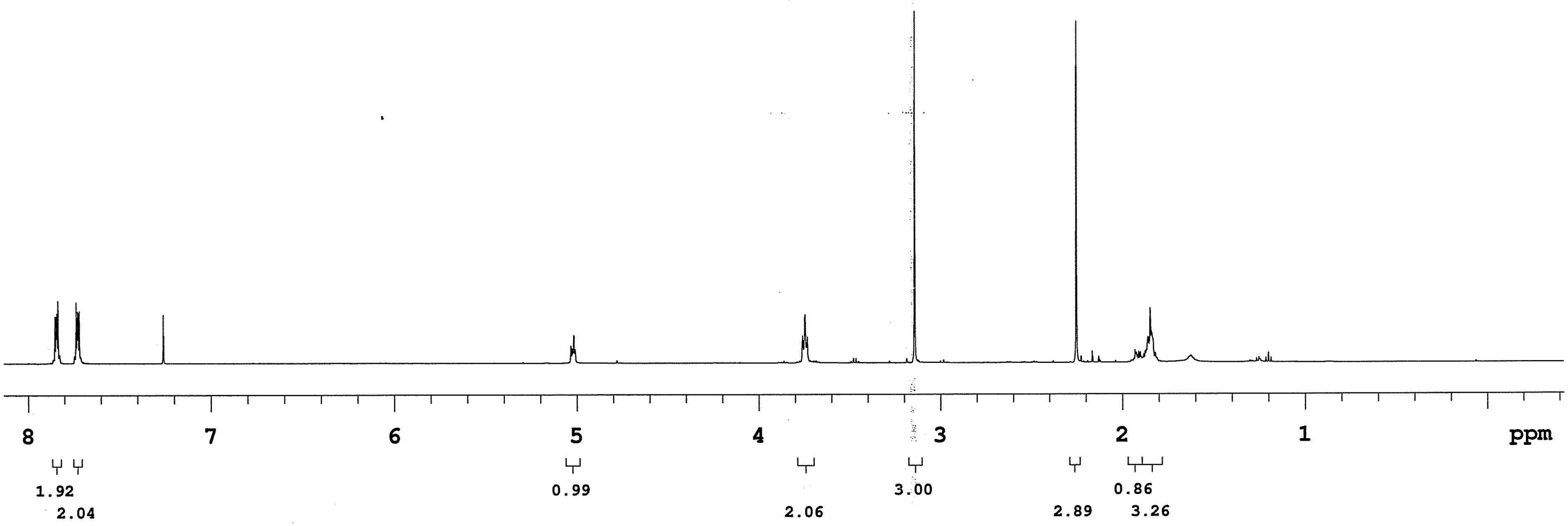

lyd-1-208-1-c

File: nmr400/Zhang/yluo/lyd-1-208-1-c.fid

Pulse Sequence: s2pul

Solvent: cdcl3
Ambient temperature
Operator: yluo
File: lyd-1-208-1-c
INOVA-500 "nmrserver"

Relax. delay 1.300 sec
Pulse 53.8 degrees
Acq. time 1.000 sec
Width 28040.7 Hz
704 repetitions
OBSERVE C13, 100.5671761 MHz
DECOUPLE H1, 399.9506784 MHz
Power 40 dB
continuously on
WALTZ-16 modulated
DATA PROCESSING
Line broadening 1.0 Hz
FT size 65536
Total time 6 hr, 24 min, 59 sec

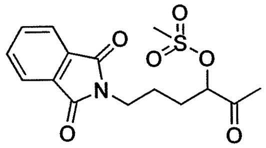

2h

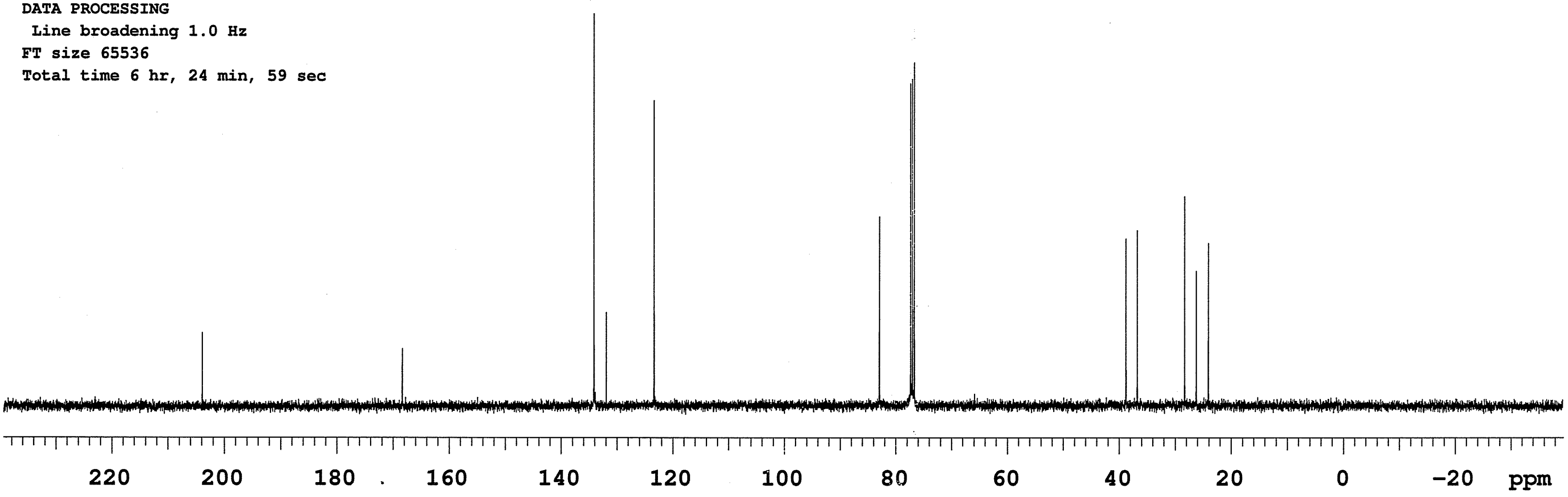

lyd-1-206-4

File: nmr500/Zhang/yluo/lyd-1-206-4.fid

Pulse Sequence: s2pul

Solvent: cdcl3
Ambient temperature
Operator: yluo
File: lyd-1-206-4
INOVA-500 "nmrserver"

Relax. delay 4.800 sec
Pulse 74.9 degrees
Acq. time 2.500 sec
Width 8002.4 Hz
16 repetitions
OBSERVE H1, 499.8560505 MHz
DATA PROCESSING
Line broadening 0.2 Hz
FT size 32768
Total time 0 min, 0 sec

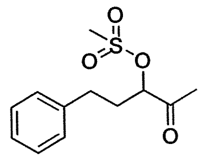

2i

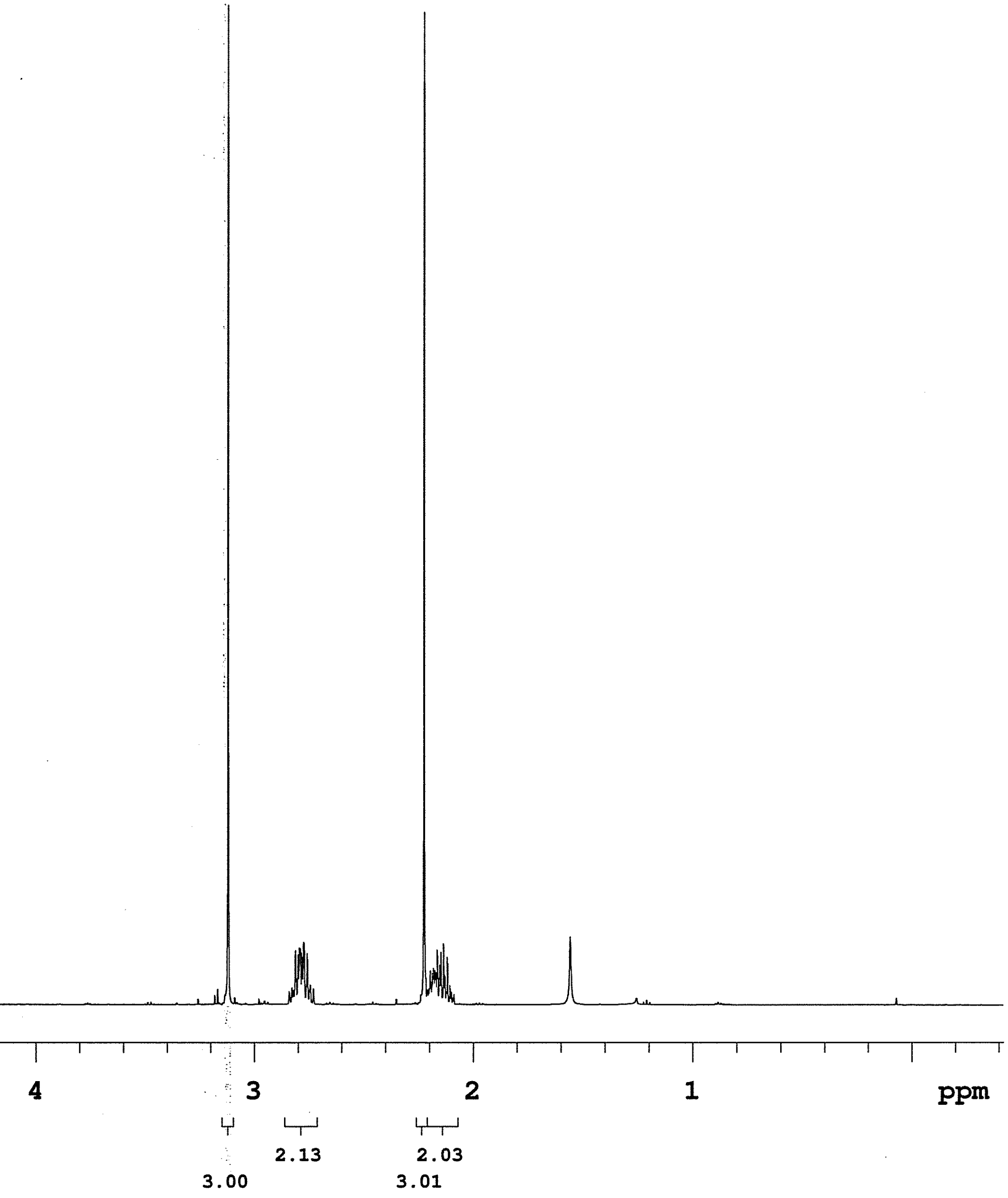

lyd-1-206-4-c

File: nmr400/Zhang/yluo/lyd-1-206-4-c.fid

Pulse Sequence: s2pul

Solvent: cdcl3
Ambient temperature
Operator: yluo
File: lyd-1-206-4-c
INOVA-500 "nmrserver"

Relax. delay 1.300 sec
Pulse 53.8 degrees
Acq. time 1.000 sec
Width 28040.7 Hz
1164 repetitions
OBSERVE C13, 100.5671761 MHz
DECOUPLE H1, 399.9506784 MHz
Power 40 dB
continuously on
WALTZ-16 modulated
DATA PROCESSING
Line broadening 1.0 Hz
FT size 65536
Total time 7 hr, 41 min, 56 sec

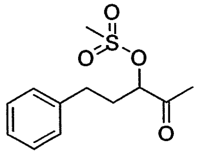

2i

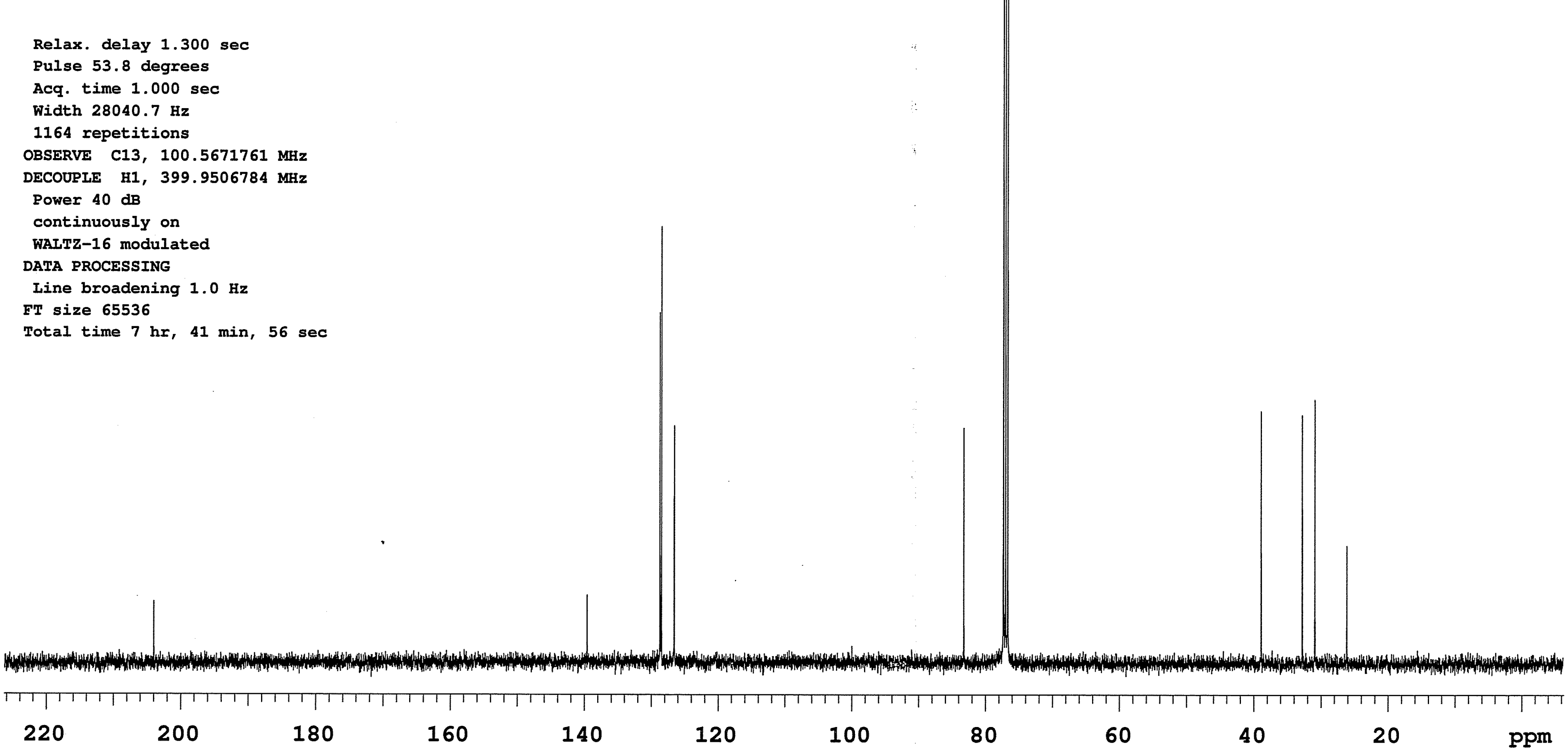

lyd-1-208-3

File: nmr500/Zhang/yluo/lyd-1-208-3.fid

Pulse Sequence: s2pul

Solvent: cdcl3
Ambient temperature
Operator: yluo
File: lyd-1-208-3
INOVA-500 "nmrserver"

Relax. delay 4.800 sec
Pulse 74.9 degrees
Acq. time 2.500 sec
Width 8002.4 Hz
28 repetitions
OBSERVE H1, 499.8560510 MHz
DATA PROCESSING
Line broadening 0.2 Hz
FT size 32768
Total time 0 min, 0 sec

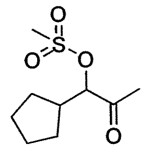

2j

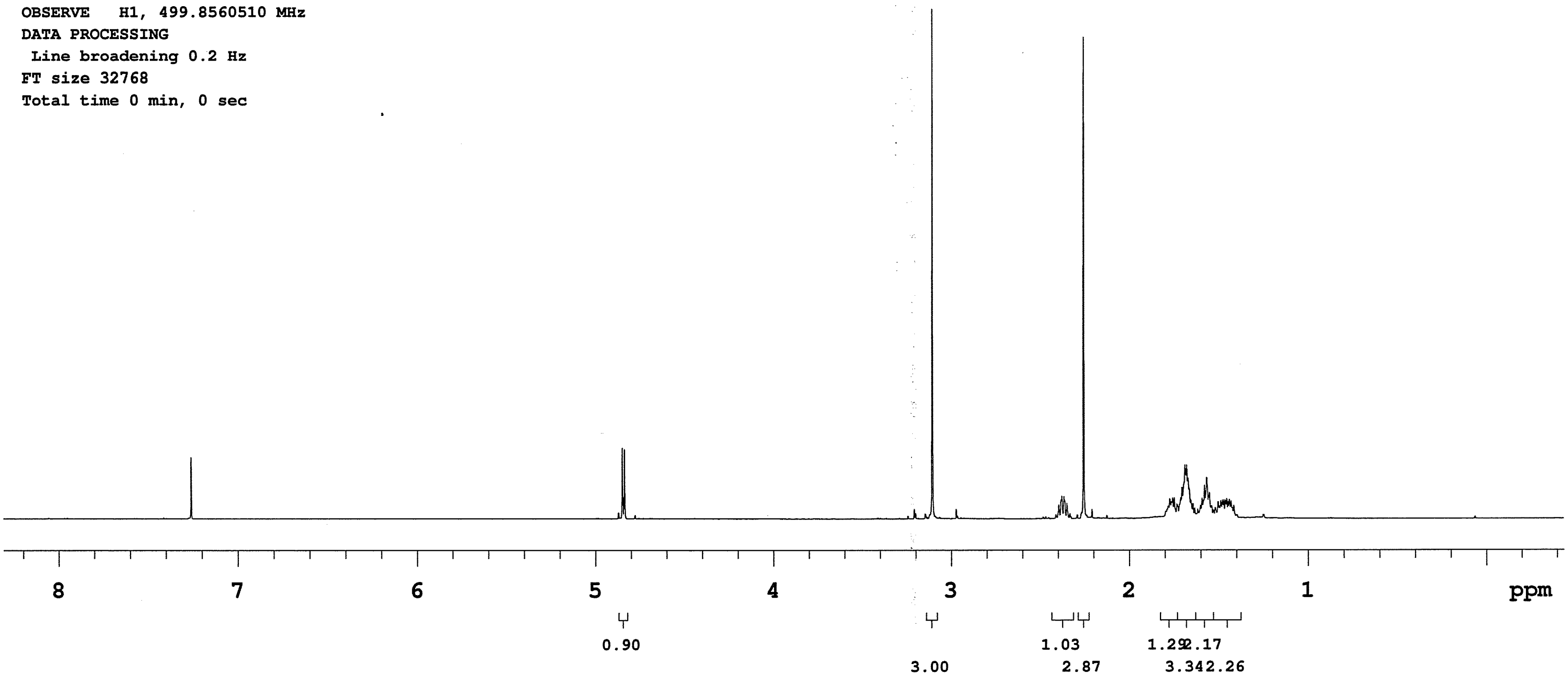

lyd-1-208-3-c

File: nmr400/Zhang/yluo/lyd-1-208-3-c.fid

Pulse Sequence: s2pul

Solvent: cdcl3
Ambient temperature
Operator: yluo
File: lyd-1-208-3-c
INOVA-500 "nmrserver"

Relax. delay 1.300 sec
Pulse 53.8 degrees
Acq. time 1.000 sec
Width 28040.7 Hz
976 repetitions
OBSERVE C13, 100.5671753 MHz
DECOUPLE H1, 399.9506784 MHz
Power 40 dB
continuously on
WALTZ-16 modulated
DATA PROCESSING
Line broadening 1.0 Hz
FT size 65536
Total time 6 hr, 24 min, 59 sec

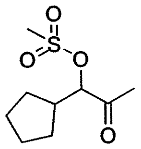

2j

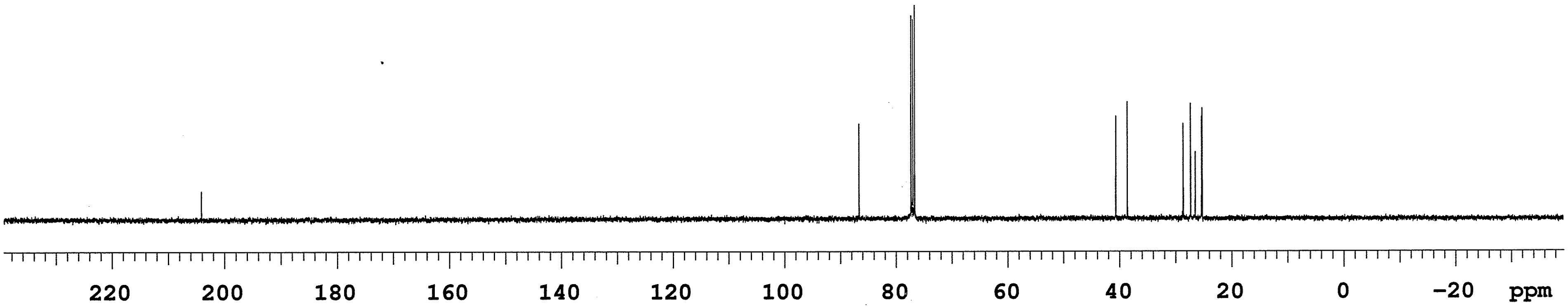

lyd-1-207-3

File: nmr500/Zhang/yluo/lyd-1-207-3.fid

Pulse Sequence: s2pul

Solvent: cdcl3
Ambient temperature
Operator: yluo
File: lyd-1-207-3
INOVA-500 "nmrserver"

Relax. delay 4.800 sec
Pulse 74.9 degrees
Acq. time 2.500 sec
Width 8002.4 Hz
12 repetitions
OBSERVE H1, 499.8560510 MHz
DATA PROCESSING
Line broadening 0.2 Hz
FT size 32768
Total time 0 min, 0 sec

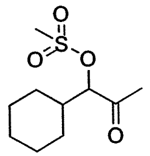

2k

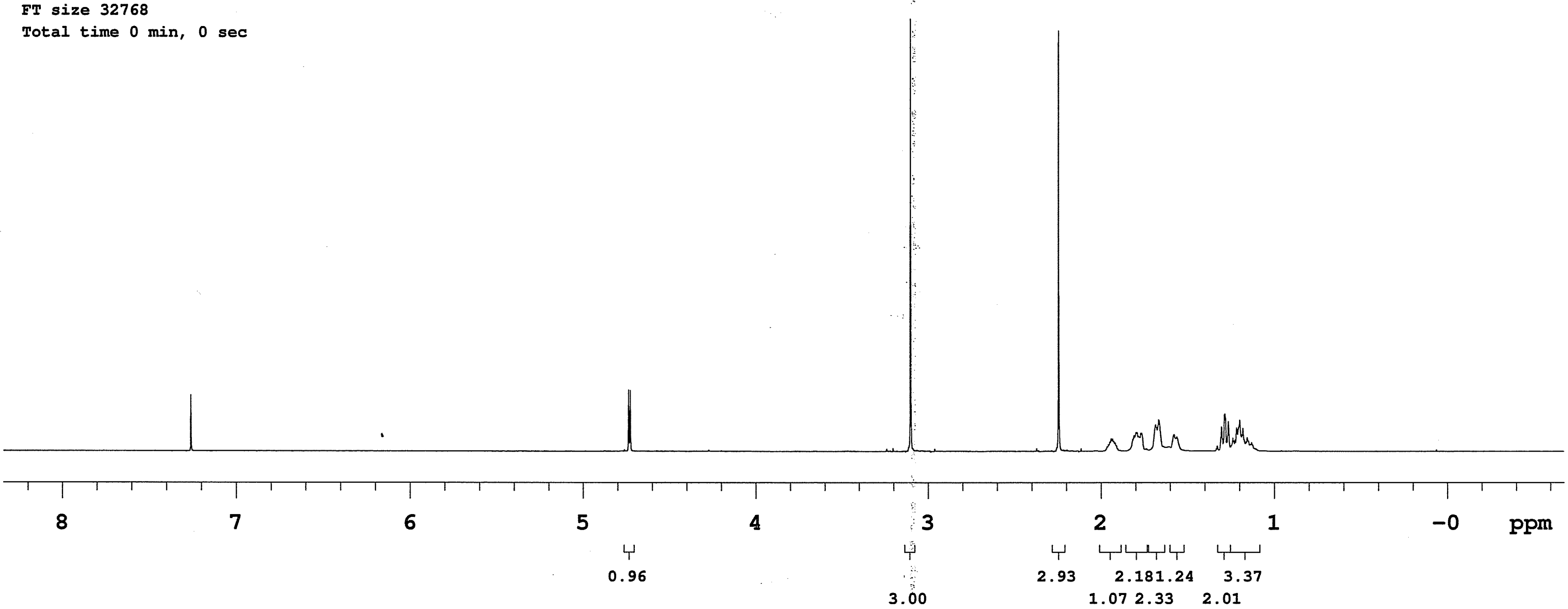

lyd-1-207-3-c

File: nmr400/Zhang/yluo/lyd-1-207-3-c.fid

Pulse Sequence: s2pul

Solvent: cdcl3
Ambient temperature
Operator: yluo
File: lyd-1-207-3-c
INOVA-500 "nmrserver"

Relax. delay 1.300 sec
Pulse 53.8 degrees
Acq. time 1.000 sec
Width 28040.7 Hz
948 repetitions
OBSERVE C13, 100.5671761 MHz
DECOUPLE H1, 399.9506784 MHz
Power 40 dB
continuously on
WALTZ-16 modulated
DATA PROCESSING
Line broadening 1.0 Hz
FT size 65536
Total time 7 hr, 41 min, 56 sec

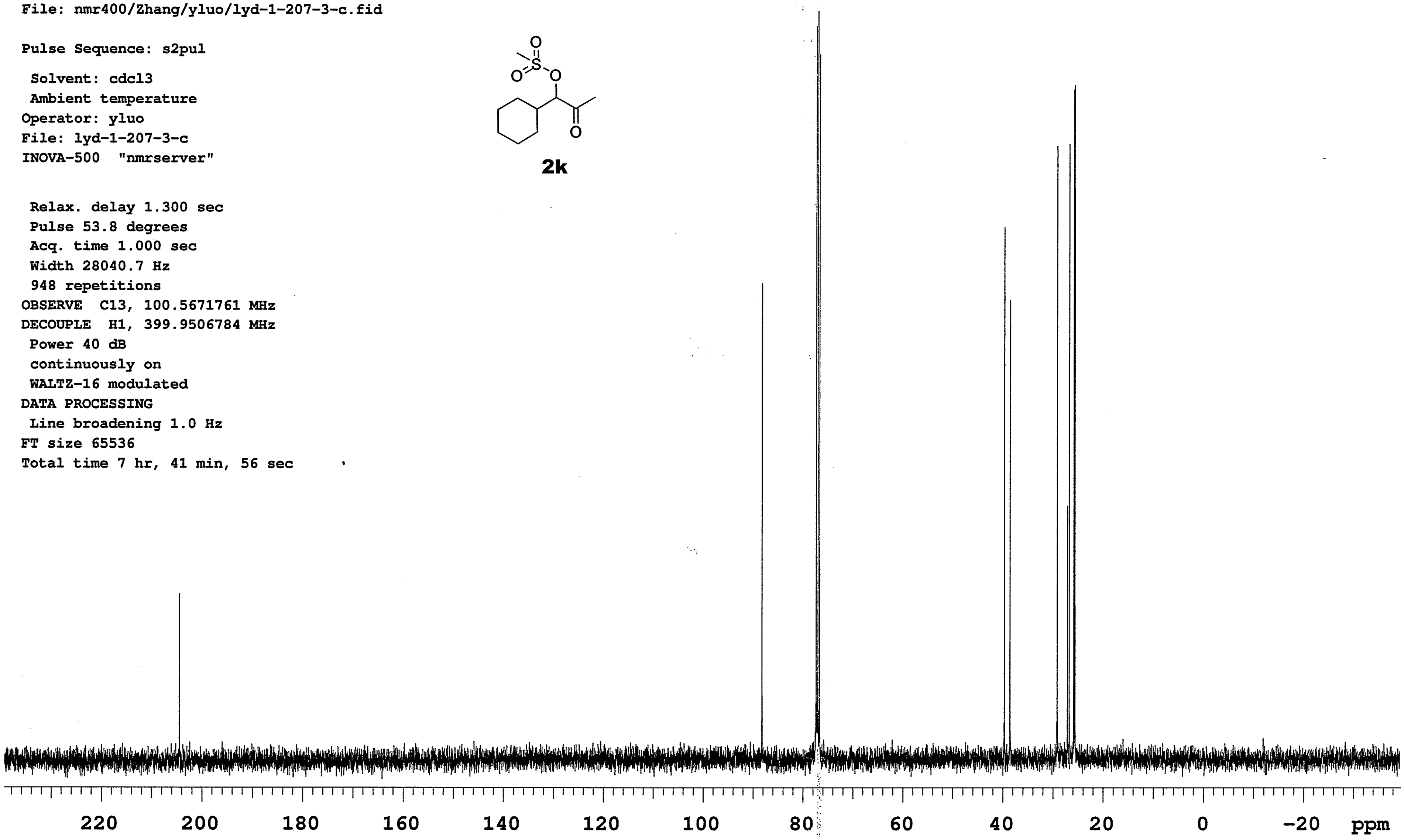

lyd-1-228-0309

File: nmr400/Zhang/yluo/lyd-1-228-0309.fid

Pulse Sequence: s2pul

Solvent: cdcl3
Ambient temperature
Operator: yluo
File: lyd-1-228-0309
INOVA-500 "nmrserver"

Relax. delay 4.800 sec
Pulse 75.0 degrees
Acq. time 2.500 sec
Width 8002.4 Hz
8 repetitions
OBSERVE H1, 399.9486723 MHz
DATA PROCESSING
Line broadening 0.2 Hz
FT size 65536
Total time 14 min, 39 sec

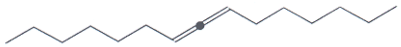

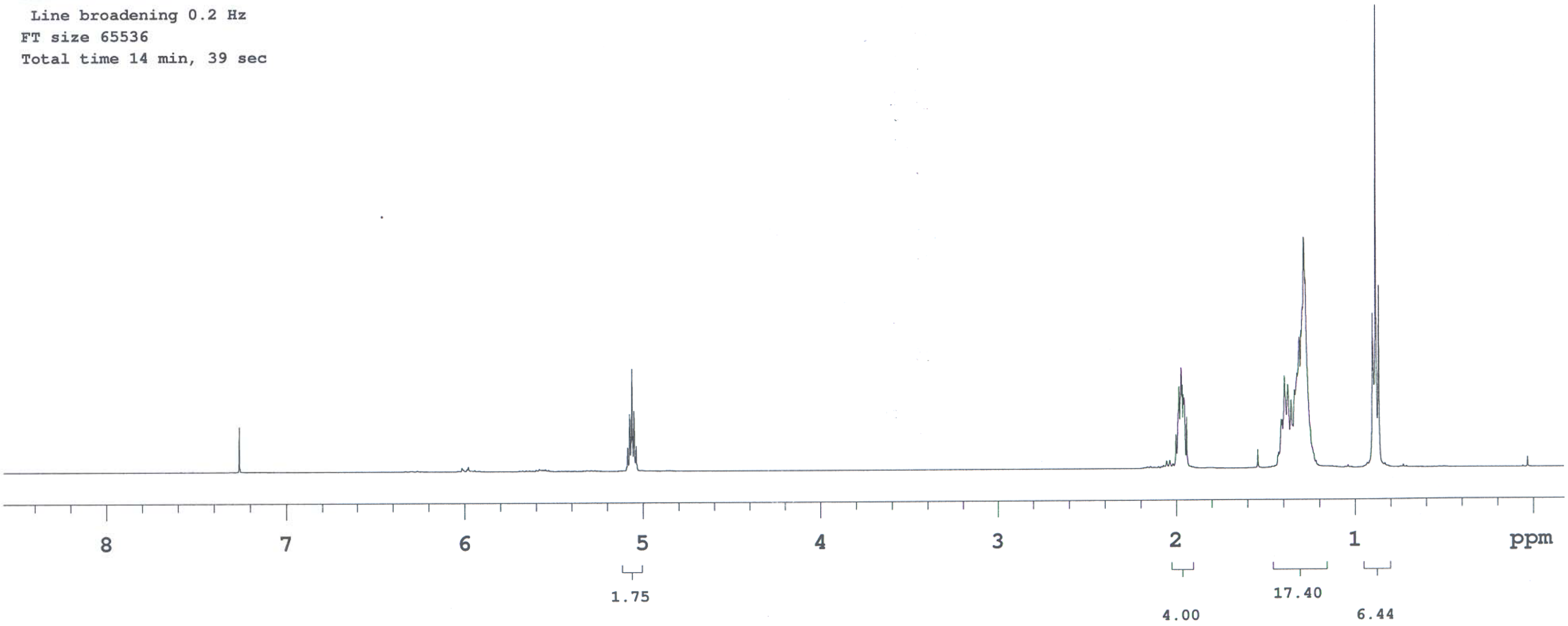

lyd-1-228-C

File: nmr400/Zhang/yluo/lyd-1-228-C.fid

Pulse Sequence: s2pul

Solvent: cdcl3
Ambient temperature
Operator: yluo
File: lyd-1-228-C
INOVA-500 "nmrserver"

Relax. delay 1.300 sec
Pulse 53.8 degrees
Acq. time 1.000 sec
Width 28040.7 Hz
24 repetitions
OBSERVE C13, 100.5671736 MHz
DECOUPLE H1, 399.9506784 MHz
Power 40 dB
continuously on
WALTZ-16 modulated
DATA PROCESSING
Line broadening 1.0 Hz
FT size 65536
Total time 6 hr, 24 min, 59 sec

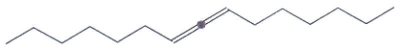

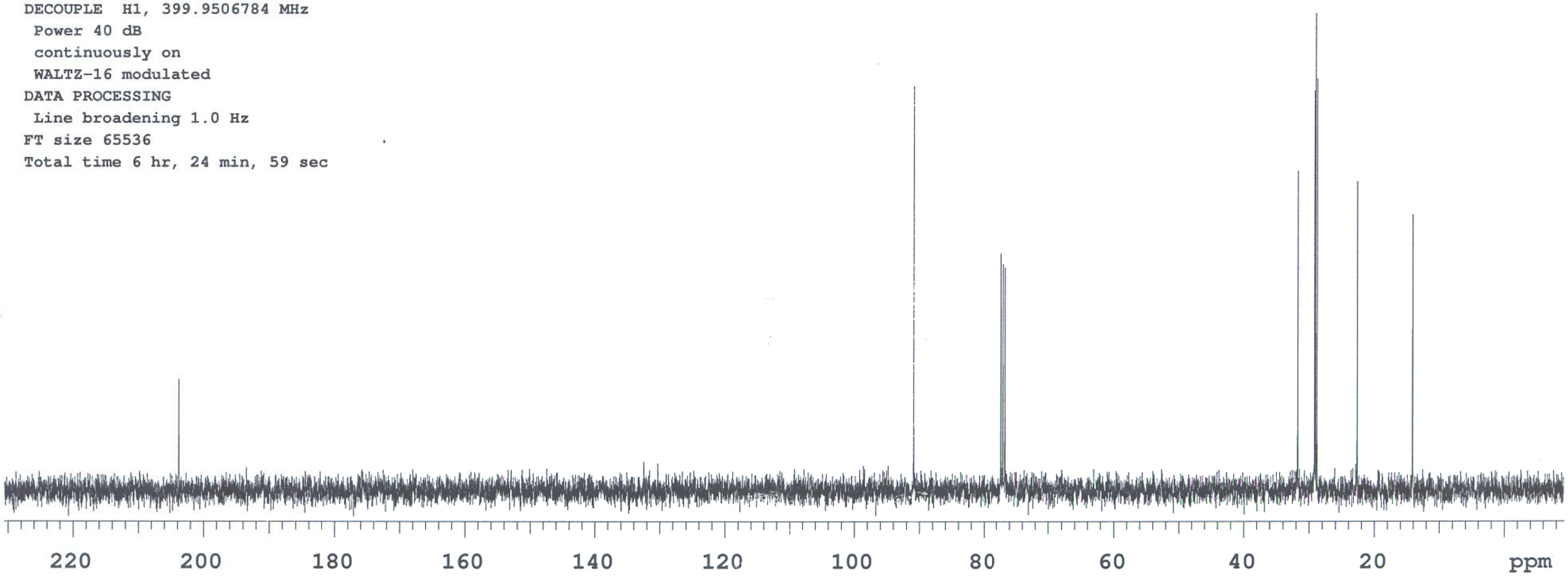

Supplement: File 2 — NMR spectra of compounds. [file Beilstein_J_Org_Chem-07-596-s002.pdf]
